# Topology-optimized dielectric cavities for enhanced excitonic light emission from WSe_2_

**DOI:** 10.1038/s41377-026-02436-w

**Published:** 2026-10-13

**Authors:** Owen Matthiessen, Brandon Triplett, Omer Yesilyurt, Davide Cassara, Karthik Pagadala, Morris M. Yang, Andres E. Llacsahuanga Allcca, Hamza Ather, Colton Fruhling, Abhishek Bharatbhai Solanki, Yong P. Chen, Hadiseh Alaeian, Alexander V. Kildishev, Vladimir M. Shalaev, Federico Capasso, Alexandra Boltasseva, Vahagn Mkhitaryan

**Affiliations:** 1 Elmore Family School of Electrical and Computer Engineering, Purdue University, West Lafayette, IN 47906 USA; 2 Birck Nanotechnology Center, Purdue University, West Lafayette, IN 47906 USA; 3 John A. Paulson School of Engineering and Applied Sciences, Harvard University, Cambridge, MA 02138 USA; 4 Department of Physics and Astronomy, Purdue University, West Lafayette, IN 47907 USA; 5 Purdue Quantum Science and Engineering Institute, Purdue University, West Lafayette, IN 47907 USA

**Keywords:** Sub-wavelength optics, Micro-optics

## Abstract

Photonic inverse design and, especially, topology optimization, enable dielectric cavities with deeply sub-diffraction mode volumes and high quality factors, thus offering a powerful platform for enhanced light-matter coupling. Here, we design and fabricate arrays of CMOS-compatible silicon cavities on sapphire with extreme subwavelength transverse mode sizes of only 30–40 nm (${{\bf{V}}}{{\boldsymbol{\sim }}}{{{\boldsymbol{\lambda }}}}^{{{\boldsymbol{3}}}}/{{\boldsymbol{2500}}}$). These cavities are engineered for deterministic coupling to a monolayer (or few-layer) excitonic material, producing strong near-field localization directly beneath the 2D material. Photoluminescence (PL) measurements show reproducible tenfold raw enhancement relative to bare silicon, which corresponds to a “per-area” normalized enhancement on the order of 10^3^, accounting for the small area of the deeply subwavelength mode volume, consistent with numerical simulations that account for material absorption and fabrication tolerances. Furthermore, time-resolved PL measurements reveal pronounced lifetime shortening and non-exponential dynamics, indicating cavity-mediated exciton-exciton interactions. The optimized cavity geometry enhances the far-field collection efficiency and supports scalable integration with van der Waals semiconductors. Our results show that the arrays of topology-optimized dielectric cavities are a versatile, scalable platform for controlling excitonic emission and interactions, which creates new opportunities in nonlinear optics, optoelectronics, and quantum photonics.

**Figure:**
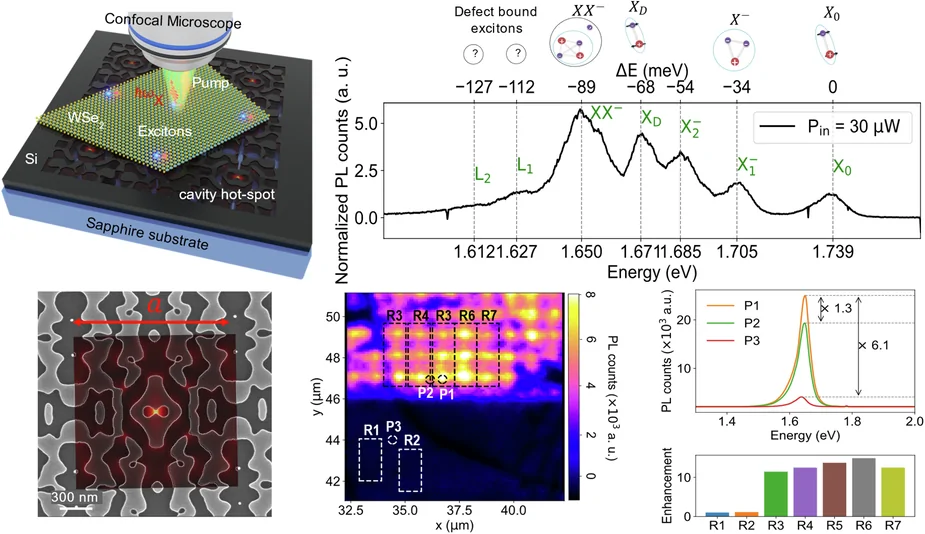

## Introduction

Strong and tailorable light-matter interaction at the nanoscale is essential for modern photonics. It enables on-chip quantum and nonlinear optics, low-threshold lasing, and efficient photon sources. Achieving this regime requires efficiently confining electromagnetic energy to modes with small mode volumes and high quality factors (Q-factors). Plasmonic nanostructures have long enabled extreme subwavelength confinement and Purcell enhancement^1,2^, offering the potential to greatly accelerate radiative processes^3,4^ and overcome decoherence^5^ in semiconductor quantum dots and other solid state and molecular emitters^2,4^ as well as 2D materials^6–16^. However, they are fundamentally limited by ohmic losses. In quantum applications, these losses can cause photons generated by quantum emitters to be reabsorbed by plasmons, reducing efficiency and coherence. All-dielectric nanophotonic cavities provide a compelling alternative^17–25^. They combine low loss with compatibility for large-scale photonic integration. The performance of conventional dielectric cavities, however, is often constrained by a large mode volume. In such traditional architectures, such as photonic crystals^26–29^, microring resonators^30–33^, and Distributed Bragg Reflector cavities^34–37^, this constraint restricts field localization to the diffraction-limited volume and results in weaker emitter-cavity coupling.

Recent advances in photonic inverse design and topology optimization have led to a new class of dielectric and plasmonic cavities that support optical modes with extreme field enhancement and compact mode volumes^38–48^. For dielectric structures, this approach exploits the full design freedom of computational optimization to concentrate light into deep-subwavelength regions, achieving mode volumes comparable to plasmonic systems but with negligible absorption losses. Initially applied to optimize grating couplers^43,49,50^, topology optimization has since evolved to incorporate fabrication constraints in the inverse design of plasmonic and dielectric nanophotonic devices^39,40,51,52^. Early implementations in plasmonic structures have improved field confinement and directional emission from quantum emitters^42,53–55^, while more recent efforts have shifted toward low-loss dielectric platforms; using silicon^56^ or group III-V materials such as gallium (GaP)^41^ and indium phosphide (InP)^57^. These advances have enabled experimental demonstrations in nanolasing^46,47^, enhanced fluorescence^42^, strong coupling with monolayer transition metal dichalcogenides (TMDC) at cryogenic temperatures^48^, nonlinear optical enhancement^57^, and efficient light-matter interactions^58,59^.

Two-dimensional van der Waals semiconductors, such as WSe_2_ and other TMDCs, offer exciting opportunities for photonics and quantum photonics applications. They host tightly bound excitons with large oscillator strengths and are compatible with a wide range of substrates. These materials are ideal platforms for exploring rich exciton and exciton-polariton physics^60^. Applications range from photodetection^61,62^ and light emission^30,63^ to single-photon-level nonlinearities^48,64^, valleytronics^65–67^, and solid-state quantum simulation^68,69^. In recent years, they have been the focus of intense research on various types of excitons^70,71^ and exciton complexes^72–75^ in both monolayers^73,76^ and Moiré heterostructures^68,69,77–79^. However, their atomic thickness limits the volume for interaction with free-space optical fields, requiring engineered photonic environments to boost light-matter coupling.

In this work, we explore the coupling of a WSe_2_ monolayer to 2D-periodic arrays of identical topology-optimized silicon dielectric cavities fabricated on sapphire substrates. A deeply subwavelength gap in the center of each inverse-designed cavity enables strong localization of optical fields directly beneath the monolayer. This approach provides a straightforward way to create deterministic hotspots while retaining control over nanometer-scale light confinement. Such capability is advantageous compared to plasmonic gap modes, which achieves extreme field enhancements but lacks controlled placements of chemically synthesized nanoparticles. The decision to use topology-optimized cavities was motivated by their distinctive advantage: they can achieve extremely strong field localization while remaining in a low-loss dielectric platform. This combination is especially relevant for emerging quantum photonic applications, where strong enhancement must be realized without introducing dissipative losses. Importantly, while topology-optimized dielectric cavities with extreme field confinement have already been demonstrated experimentally, coupling realistic excitonic materials to such cavities has not been extensively reported. An experimental demonstration of this integration and the resulting enhancement of light-matter coupling can open new avenues for studying and controlling excitons, including exciton trapping, exploration of single-exciton-single-photon nonlinearities, and other related phenomena.

Numerical simulations of the studied topology-optimized cavity arrays reveal strong optical field confinement in nanoscale hotspots at the center of each cavity, with near-field amplitude raw enhancements exceeding 50× relative to the incident field under realistic material losses in silicon and absorption by the WSe_2_ monolayer (for detailed discussion, see Section). Additionally, the dipole emission simulations show a marked increase in the emission directionality when the emitter is aligned with the linearly polarized bowtie-like mode of the cavity, compared to emission from WSe_2_ on flat, unpatterned silicon.

Experimentally, we observe a consistent raw photoluminescence enhancement of approximately 10× when comparing off-cavity regions, confirmed by both spatial PL maps and individual spectra. Accounting for the extreme field confinement by normalizing to the effective hotspot area yields a normalized enhancement of order ∼10^3^. Throughout this work, “raw enhancement” refers to the directly measured PL intensity ratio, while “normalized enhancement” accounts for the effective hotspot area or mode volume. We want to emphasize that the normalization used in the literature, and adopted here, is intended solely to facilitate comparisons across studies. We believe that the reported “raw enhancement” values are more physically meaningful for practical applications. Nevertheless, we include the normalized enhancement to enable direct comparison with prior works, which typically report enhancement in this normalized form. Accordingly, we compiled Table I to provide both raw and normalized enhancement values for our work as well as for the references cited therein.

Low-temperature PL spectroscopy further reveals a rich manifold of excitonic states, including bright excitons, dark excitons, and biexcitons seen previously in other studies^73,80–86^ (see Supplementary Fig. S1). With metasurface cavities, these features are more defined than in flat regions of the planar substrate, resulting in narrower linewidths. This behavior is consistent with cavity-assisted Purcell enhancement of the radiative decay rates and diminished contribution from non-radiative pathways.

Furthermore, time-resolved PL measurements reveal that the exciton decay dynamics become substantially non-exponential in the presence of the cavity in the entire range of pump powers. In contrast, the decay remains nearly exponential over a broad range of pump powers in the absence of the cavity, deviating only at the higher powers. The observed difference between on- and off-cavity behavior, together with its power dependence, points to the onset of multi-exciton interactions and cavity-enhanced energy transfer processes^87–91^.

These observations highlight the potential of topology-optimized dielectric metasurface cavities for controlling emission processes and excitonic interactions in atomically thin semiconductors, as well as for probing nonlinear and many-body phenomena in strongly confined optical environments.

## Results

In this study, we use photoluminescence (PL) spectroscopy to investigate the interaction of excitons in WSe_2_ monolayers (MLs) and topology-optimized dielectric cavities with subwavelength mode confinement. The cavities were developed by using the photonic inverse design approach, which is described in detail in the “Methods” section. The metasurface cavities consist of a 100–200 nm-thick Si cavity layer on top of a sapphire substrate. Although silicon is relatively lossy at the exciton emission wavelengths in WSe_2_, it was chosen for its availability, technological relevance, and ease of fabrication. These intrinsic losses limit the achievable quality factor; however, the high refractive index of Si enables strong field confinement within small spatial regions and allows us to achieve strong interaction between this mode and excitons. While silicon is absorptive at the operating wavelengths, it is essentially lossless in the near-infrared and is an ideal platform for such cavity applications^44^. Additionally, in our design, we deliberately used supported cavities rather than suspended membranes, as this configuration offers greater mechanical robustness and practicality for scalable integration of 2D materials, in contrast to suspended photonic devices.

**Fig. 1:**
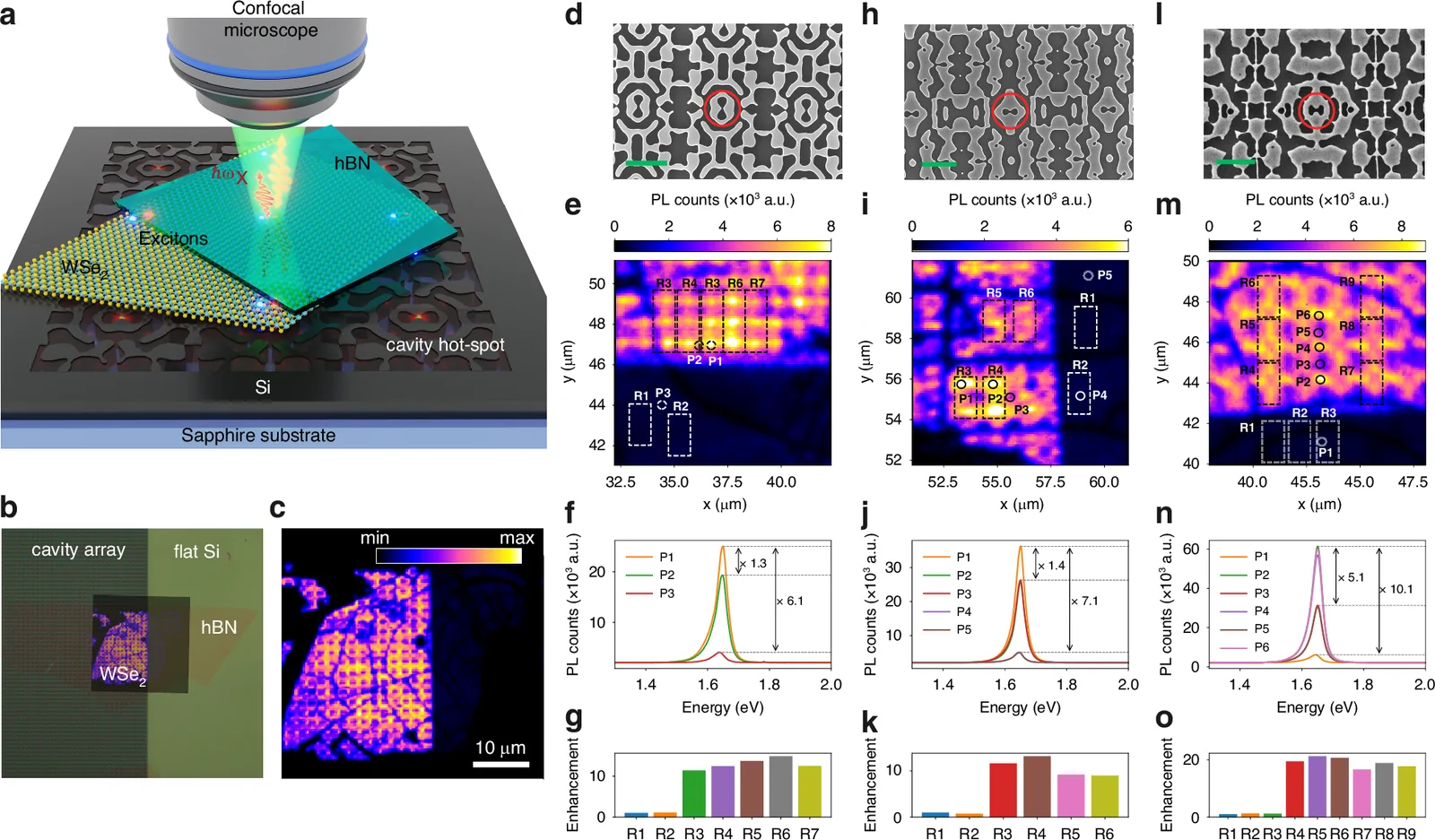
PL maps and enhancement of WSe_2_ coupled to topology-optimized cavities. **a** Illustration of the confocal microscopy setup used to collect PL maps from the hBN/WSe_2_/topology- optimized cavity/sapphire stack. In all experiments, the flakes are positioned such that part of the WSe_2_ flake lies on the cavity array and part on the unpatterned Si region, providing a reference signal. **b**, **c** Optical microscope image at the edge of a cavity array, with the inset PL map superimposed to indicate the location of the WSe_2_ flake relative to the cavity boundary since PL emission occurs predominantly from WSe_2_. The PL map is collected over a 40 × 40 µm^2^ region, covering both cavity and reference areas. **d**–**o** Data for three representative samples, including SEM images, high-resolution PL maps, PL intensity counts, and raw enhancement h**i**stograms. **e**, **i**, **m** show the PL maps of all three samples. The circular markers indicate the positions where PL spectra shown in (**f**, **j**, **n**) were measured. The dashed rectangles denote the regions used to compute the average raw PL enhancement factors shown in (**g**, **k**, **o**). The identical rectangular regions were selected on the flat Si reference and the patterned cavity arrays to ensure a consistent comparison. The scale bars on SEM images in (**d**, **h**, **l**) are all 500 nm

Large-area (typically ~ 40 × 30 μm²) hBN-capped WSe_2_ monolayers were transferred onto the cavity arrays using a gold-assisted transfer method^92^. Each flake was carefully positioned to lie on both the cavity array and on the adjacent flat Si-on-sapphire substrate, as shown in Fig. 1a, allowing for a direct on-chip comparison between cavity-coupled and reference (uncoupled) emission. In the following, we refer to regions of the WSe_2_ flake located on top of the cavity arrays as “on-cavity”, while the regions located on the adjacent unpatterned Si/sapphire substrate are referred to as “off-cavity”. After the transfer, the strain on the flakes, which is induced by proximity to the cavity was characterized using Raman spectroscopy. We found that the strain inhomogeneity is negligible, with Raman peak shifts of less than 1 cm^−1^ compared to the reference region of the flake on the flat Si/sapphire substrate. A detailed discussion of the Raman maps and shift analysis is provided in Supplementary Fig. S2. Figure 1b shows an example optical microscope image of an hBN/WSe_2_ flake positioned over one of the cavity arrays studied in our experiments. The image includes the PL map from Fig. 1c overlaid to indicate the location of the WSe_2_ flake beneath the hBN encapsulation. The PL map in Fig. 1c clearly reveals the enhanced emission from the cavity region compared with the weaker response observed from the off-cavity reference. We studied three different topology-optimized metasurface cavities (Fig. 1d–o) that were obtained through the same optimization process but with different random initiations of the algorithm, with the important exception that the cavity in Fig. 1l which was optimized for the case when WSe_2_ is present. Our optimization process consistently converges to designs featuring a bowtie-like structure in the middle of the unit cell, as shown in SEM images in Fig. 1d, h, l with the red outlines. Such consistent appearance of the bowties in all devices indicates the presence of a global minimum with this feature, while variations in the surrounding dielectric distribution correspond to nearby local minima to which the algorithm converges.

The panels in Fig. 1d–f show scanning electron microscopy (SEM) images (Fig. 1d), high-resolution PL map (Fig. 1e), integrated PL spectra over 10 s (Fig. 1f), and the histograms of raw PL enhancement factors (Fig. 1g) of our first cavity. The circular markers in Fig. 1e indicate the locations of the points for which the spectra are shown in panel Fig. 1f. The arrows in panel Fig. 1f highlight the relative enhancement of peak counts for different positions on the cavity. For this specific cavity, we see more than 6 times raw enhancement of the PL peak counts compared to the off-cavity region. Figure 1g shows the average raw PL enhancement, obtained by comparing the mean PL counts from identically sized rectangular regions on and off the cavity arrays in the PL maps of Fig. 1e. For this sample, the analysis yields the raw enhancement of approximately 14 times. Figure 1h–k and Fig. 1l–o present the equivalent measurements and analyses for the two other cavity arrays with different geometries. Among those, the cavity shown in Fig. 1l–o exhibits the largest enhancement- nearly twice of what is observed for the other two arrays. We want to emphasize that the enhancement factors in Fig. 1f, j, n are calculated with respect to point P1 which is in the off-cavity region for all the samples. These differences can be partly explained by the resonance shifts relative to the exciton emission wavelength, arising from fabrication tolerances, as well as variations in the observed collection efficiency, since some cavities provide superior emission directionality compared to others (see the discussion below). The combined effect of these factors leads to the observed variations in enhancement across different cavities in our experiments.

**Fig. 2:**
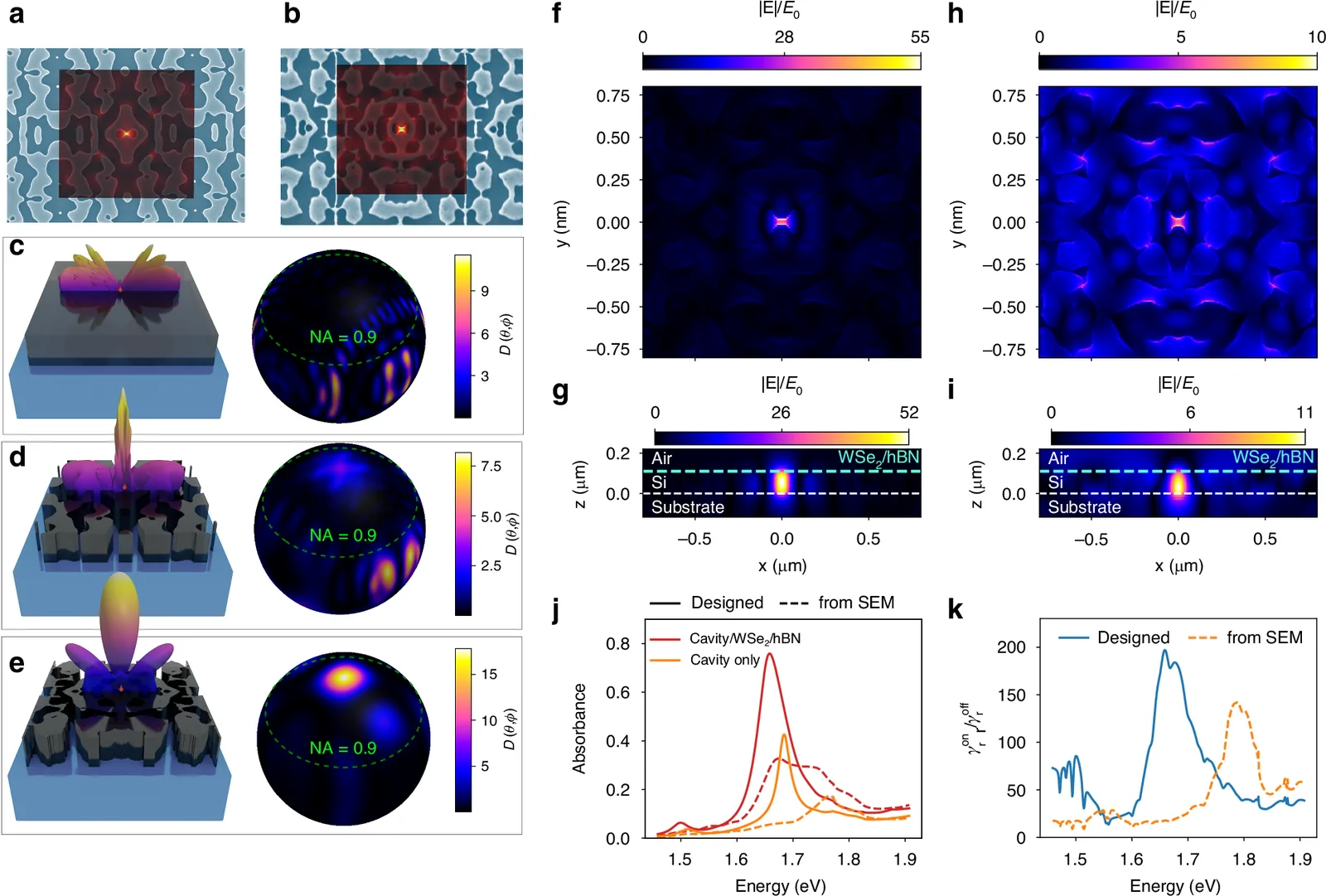
Simulation results for topology-optimized dielectric cavities. **a**, **b** The simulated near-field distributions overlaid on two example cavity designs, highlighting the locations of localized hotspots. **c** Far-field radiation pattern (left) and radiation directivity plot (right) for a dipole placed off the cavity. The green dashed line indicates the collection numerical aperture (NA) of the microscope objective. **d**, **e** Same as (**c**), but for the two topology-optimized cavities shown in (**a**) and (**b**), respectively. **f**, **g** The simulated electric near-field distributions in the xOy and xOz planes for a unit cell of the cavity design in (**b**) as intended in the simulat**i**on. **h**, **i** Same as (**f**, **g**), but using the pattern contours extracted from the experimental SEM images. These results incorporate fabrication distortions and illustrate their impact on near-field enhancement. **j** the simulated absorbance spectra of the cavity design in (**b**), with and without the WSe_2_/hBN stack, shown both for the ideal design (the solid lines) and the SEM-extracted structures (the dashed lines). The fabricated cavities exhibit a blue shift relative to the design and a red shift when loaded with the WSe_2_/hBN stack. **k** the simulated radiative emission rate enhancement relative to the off-cavity case, for the ideal and SEM-extracted structures

We performed numerical simulations for the designed cavities shown in Fig. 1. These include studies of the near-field distributions, far-field radiation patterns, and the resonance spectra (Fig. 2). The simulated near-field distributions of a cavity array unit cell overlaid on the scanning electron microscopy (SEM) images (Fig. 2a, b) clearly reveal the strong localization of the cavity mode within the bowtie-like gap region at the cavity center. Figure 2c–e display the far-field radiation patterns (left panel) and the corresponding directivity plots (right panel) for a dipole emitter placed on the off-cavity region (Fig. 2c) and at the center of bow-tie like regions of two representative cavities in Fig. 2d and Fig. 2e. In all cases, the dipole is aligned with the cavity mode polarization and positioned at the top surface plane of the structure. The simulations incorporate the full material stack, including the WSe_2_ monolayer and the encapsulating hBN layer, with the precise hBN thickness values determined from atomic force microscopy (AFM) measurements (see Supplementary Fig. S3). The dipole radiation pattern plots (Fig. 2c–e) reveal that a dipole placed on the off-cavity region primarily emits at grazing angles relative to the surface normal. However, a dipole of the same polarization placed in the ‘hot-spot’ region at the top of the cavity surface exhibits highly directional emission. The significant fraction of the radiated power for this case is confined within the numerical aperture (NA) of our collection objective (NA = 0.9). The radiation directivity ${{\rm{D}}}\left({{\rm{\theta }}},{{\rm{\phi }}}\right)$ in Fig. 2c–e is defined as $D\left({{\rm{\theta }}},{{\rm{\phi }}}\right)=\frac{4{{\rm{\pi }}}I\left({{\rm{\theta }}},{{\rm{\phi }}}\right)}{{P}_{{\mbox{tot}}}}$, where ${{\rm{I}}}\left({{\rm{\theta }}},{{\rm{\phi }}}\right)$ is the radiated intensity in direction $\left({{\rm{\theta }}},{{\rm{\phi }}}\right)$ and ${P}_{{\mbox{tot}}}$ is the total radiated power. It quantifies how directional the emission is compared to an isotropic radiator (${{\rm{D}}}=1$).

The near-field distributions were simulated for two cross-sections (Fig. 2f, g): the x-y plane at the position of the WSe_2_ monolayer (Fig. 2f), and the x-z plane across the cavity at ${{\rm{y}}}=0$ (Fig. 2g) for the cavity shown in Fig. 1l. These near-field distributions are extracted at the exciton emission energy of 1.65 eV. From simple geometrical estimates of these simulated near-field distributions, We determine the effective mode volume to be approximately ${V}_{{\mbox{eff}}}\approx 30\times 30\times 100{{\mbox{nm}}}^{3}$. For the ideal cavity geometry, the simulations predict up to a 50-fold raw enhancement of the local field intensity. Figure 2h, i show the near-field distributions for the same cavity design but with the contours extracted from the experimental SEM images (see Supplementary Fig. S4). In our experiments, however, the small feature sizes and high pattern density increase the proximity effects in electron beam lithography (EBL) producing slight shape distortions from the original design. Combined with the roughness introduced during reactive-ion etching, these deviations increase radiative losses, reducing the field localization, and limit the attainable enhancement. These effects are especially pronounced for cavity resonances at shorter wavelengths, where required feature sizes shrink and fabrication tolerances tighten proportionally. We show that the near-field raw intensity enhancement is reduced to approximately 10-fold (Fig. 2h), in agreement with the experimentally observed raw photoluminescence enhancement and lifetime shortening. This reduction arises not only from the geometric imperfections that introduce light scattering and cause destructive interference, but also from resonance shifts and broadening resulting from integration with the WSe_2_/hBN stack. Indeed, Fig. 2j shows the simulated absorption spectra for the ideal (solid lines) and SEM-extracted (dashed lines) cavity geometries, both with and without the WSe_2_/hBN stack. The presence of WSe_2_ introduces additional resonance broadening and redshifts due to absorption and radiative losses. Fabrication imperfections further exacerbate these effects, resulting in significant degradation of the field enhancement. A similar trend is evident in the calculated radiative decay rate ratios on and off the cavity (Fig. 2k).

**Fig. 3:**
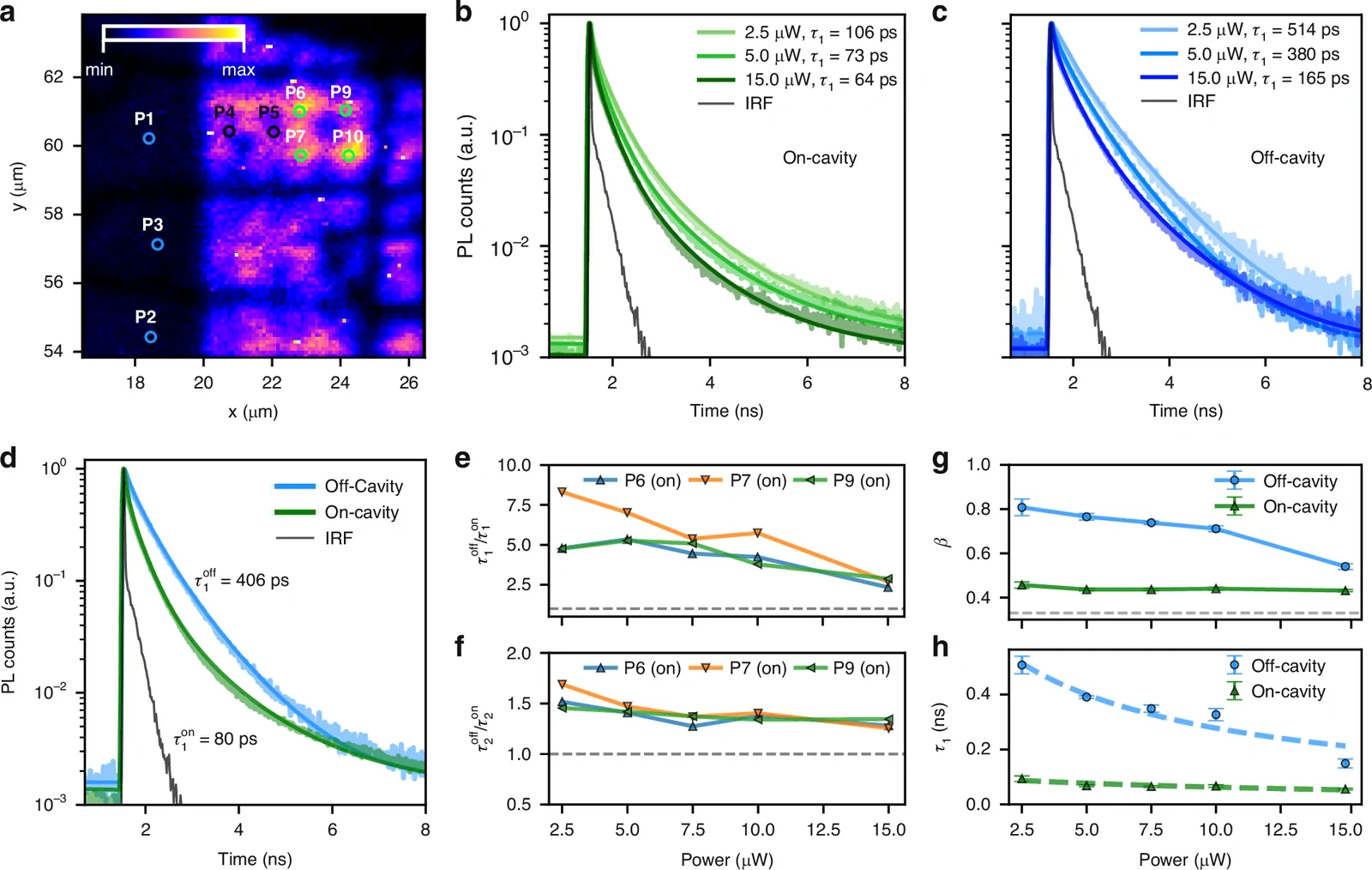
Decay dynamics of WSe_2_ excitons on and off the cavity arrays. **a** PL map showing selected measurement points on and off the cavity. **b** the decay curves (green) from a cavity location (Point 10) at varying input powers, with corresponding mixed-model fits. The gray trace shows the instrument response function (IRF). **c** the decay curves (blue) from a reference location off the cavity (Point 1), with corresponding fits and IRF as in (**b**). **d** the averaged decay curves and fits on (blue) and off (green) the cavity, demonstrating the average ×5 lifetime reduction on the cavity. **e** the ratio of short decay times ${\tau }_{1}^{{\mbox{off}}}/{\tau }_{1}^{{\mbox{on}}}$ for selected points as a function of pump power. **f** Same as (**e**), but for the long decay time ${\tau }_{2}$ from the mixed-model fits. **g** Stretching factor *β* as a function of pump power on and off the cavity. **h** the averaged short decay time ${\tau }_{1}$ on- and off- the cavity as a function of the pump power, showing a nonlinear decrease as we pump the system stronger

We further investigated the exciton decay dynamics for the on- and off-cavity cases using time-resolved photoluminescence (TRPL) measurements. These measurements were carried out using a femtosecond, pulsed (<100 fs, 80 MHz repetition rate) laser light with *λ* = 520 nm central wavelength, providing energy above the WSe_2_ band gap. Figure 3a shows PL maps with open circles indicating the locations where decay curves in Fig. 3b, c were recorded. Figure 3b, c display the decay curves for on and off the cavity cases, respectively, measured at three different pump powers: 2.5, 5, and 15 μW. These measurements reveal clear non-exponential decay dynamics. This effect is most pronounced on the cavities, where the non-exponential decays are observed at all measured pump powers (see Fig. 3b). In contrast, for off-cavity regions, such behavior appears only at higher pump powers (Fig. 3c). In both on- and off-cavity cases, we observe a clear shortening of the decay time with increasing pump power, accompanied by a more pronounced non-exponential character. This behavior suggests that the decay dynamics are more likely governed by exciton–exciton interactions, as higher pump powers generate larger exciton populations, increase their density, and thereby reduce inter-exciton distances in the pump region. Similar non-exponential decay signatures at elevated pump powers have been reported in earlier studies of WSe_2_ on unpatterned substrates, although their microscopic origin remains debated^93–95^. Depending on the experimental conditions and material quality, these types of dynamics have been attributed to a range of mechanisms, including exciton-exciton interactions^93^, defect-assisted trapping and tunneling^95^, and population transfer between bright and dark exciton states^94^. We believe that in our samples both defect-related processes and exciton–exciton interactions contribute to the observed non-exponential decay. Similar to ref. ^95^, we observe a pronounced long-time tail in the decay curves, which can be attributed to the localization effects and inhomogeneous broadening. The presence of such defects is further supported by our low-temperature spectra, where distinct low-energy emission features consistent with the defect-bound excitons are observed. At the same time, the strong power dependence of the non-exponential dynamics provides compelling evidence that exciton–exciton interactions play a dominant role, particularly in the cavity regions where the local fields significantly enhance resonant dipole-dipole interaction effects.

To further characterize the origin of the non-exponential decay dynamics, we fitted the data using several models: triple-exponential, stretched-exponentials^96^, a combined (mixed) model consisting of a stretched-exponential plus a single exponential, and the models describing the biexcitonic recombination. All fitting was performed after deconvolving each decay model with the instrument response function (IRF) to ensure physical accuracy^97^ (for details of the fitting and deconvolution procedures, see “Methods” and Supplementary Information). Among the tested models, the mixed model provides the best fit and yields consistent results for the power dependence of the decay parameters. This model is given by *I*(*t*) = *A*_1_ exp((*t/τ*_1_)^*β*^) + *A*_2_ exp(*t/τ*_2_), where the second exponential term accounts for the long-time tail of the decay curve. The stretched exponential decay curves have been widely used^88,89,91,96^, following the original proposal by Förster^87^, to describe the non-exponential dynamics in systems where the energy transfer occurs due to interactions. In this framework, the stretching parameter ${{\rm{\beta }}}$ encodes the distinct physical characteristics of the system. It depends on the dimensionality of the system and the microscopic mechanism of the interaction. Specifically, ${\rm\beta }$ is often expressed as $\beta \,=\,\frac{d}{S}$, where d is the spatial dimensionality of exciton confinement and S characterizes the type of interaction. For dipole-dipole energy transfer, ${{\rm{S}}}=6$, which is expected to be the dominant mechanism for exciton interactions at room temperature. In our case, the excitons are confined to a two-dimensional WSe_2_ monolayer, so $S\,=\,6$, leading to $\beta \,=\,\frac{1}{3}\,\approx \,0.333\left(3\right)$. Figure 3e, f show the power dependence of $\frac{{{{\rm{\tau }}}}_{1}^{{{\rm{off}}}}}{{{{\rm{\tau }}}}_{1}^{{{\rm{on}}}}}$ and $\frac{{{{\rm{\tau }}}}_{2}^{{{\rm{off}}}}}{{{{\rm{\tau }}}}_{2}^{{{\rm{on}}}}}$, respectively, extracted during this fitting. As expected, we see around ×8 lifetime shortening on-cavity compared to the off-cavity case, which is consistent with the observation of the PL enhancements (Fig. 1). At relatively low powers, the stretching exponent for the off-cavity case is ${{\rm{\beta }}}\approx 0.8$, and the decay dynamics are nearly exponential (Fig. 3g). As the pump power increases, however, *β* decreases, resulting in progressively more non-exponential decay behavior. The observed decrease of *β* with increasing excitation power suggests that the exciton-exciton interactions contribute to the non-exponential decay dynamics. At higher pump powers, the density of photoexcited carriers and excitons within the illuminated region increases, enhancing the likelihood of many-body processes such as exciton-exciton annihilation and interaction-mediated energy transfer. On the cavity, the *β* values remain very small, $\beta \,\approx \,0.4$, indicating the extreme non-exponential character of decay dynamics even at the lowest pump powers. The extracted value ${{\rm{\beta }}}\approx 0.38$ is also very close to $\beta \,\approx \,0.38$ for the expected dipole-dipole interactions. This observation further supports our earlier assumption that exciton-exciton interactions play an important role in the decay dynamics. On the cavity, the optical near fields are tightly confined within nanoscale regions, leading to substantially stronger cavity-mediated resonant exciton-exciton interactions even at relatively low pump powers.

The extracted short decay time, ${\tau }_{1}$, as a function of pump power was fitted using a power-dependent effective decay model (see SI for details), which incorporates different recombination channels through their density dependence (Fig. 3h). We find that the best agreement with the experimental data is achieved using the biexcitonic recombination model, described by

$${{{\rm{\tau }}}}_{{\mbox{eff}}}=\frac{{{{\rm{\tau }}}}_{{sp}}}{1+{{{\rm{\tau }}}}_{{sp}}{BG}{P}_{{\mbox{in}}}}$$

where G denotes the proportionality constant between the initial exciton density and the pump power (${n}_{0}=G{P}_{{\mbox{in}}}$ in the weak-excitation limit), ${\tau }_{{sp}}=\frac{1}{A}$ is the spontaneous recombination lifetime (*A* is the spontaneous recombination rate), and *B* is the biexcitonic recombination coefficient. We find that the biexcitonic recombination rate on the cavity is more than an order of magnitude larger than that of the off-cavity case (${B}_{{\mbox{on}}}/{B}_{{\mbox{off}}}\approx 33$). This result further confirms that the cavity enhances exciton–exciton interaction effects, and consequently biexcitonic recombination rates, by concentrating the optical near fields into nanoscale hotspots and thereby increasing the local exciton density in their vicinity.

**Fig. 4:**
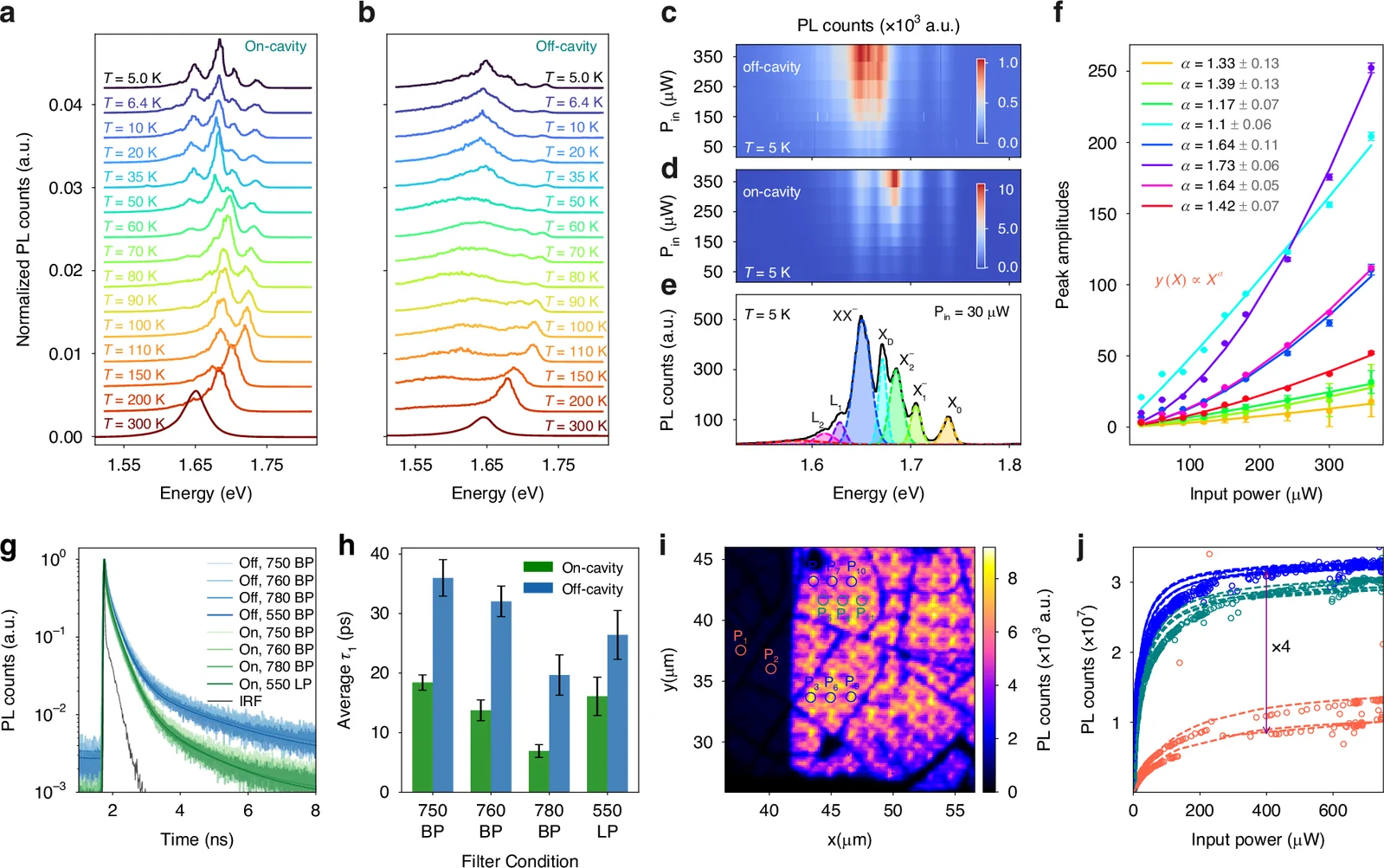
Low temperature spectra, power and temperature dependence fits. **a** on and **b** off-cavity temperature dependent WSe_2_ spectra at temperatures from *T* = 5.0 K to *T* = 200 K. **c**, **d** the power dependent density plots of WSe_2_ spectra for the off and on the cavity cases, respectively, collected at 5 K fixed temperature. At elevated excitation powers, some excitonic resonances exhibit pronounced broadening, leading to their merging with adjac**e**nt peaks. **e** the example spectra at 5 K and for input power *P*_in_ = 30 µW and their Gaussian fitting. The peak assignment is based on the comparison of the measured peak energies and their relative shifts with values reported in the literature. **f** the scatter data with the error bars are the peak amplitudes extracted from the fits as a function of the input power. The error bars represents the fit errors. The solid lines show the power low ${A}_{j}\propto {P}_{{\mbox{in}}}^{\alpha }$ fits to these data. The power α and respective fit errors are shown as a legend for each individual peak. **g** the energy filtered lifetime measurements at room temperature on and off the cavity. **h** the fitted fast decay times ${\tau }_{1}$ for the on- and off-cavity cases as extracted from the fitting of the data in (**g**). **i** the reference PL map to show the specific points for which the saturation curves in (**j**) are measured. **j** the measured saturation curves on (the blue and green scatter data points) and off the cavity (the orange points)

Another clear indication of lifetime shortening results from the comparison of the low-temperature spectra of various excitonic species for the on- and off-cavity array. Temperature-dependent PL spectra of hBN-encapsulated WSe_2_ from $T=5{\mbox{K}}$ to $T=200\,{\mbox{K}}$ for the on- and off-cavity cases show multiple excitonic peaks at cryogenic temperatures (Fig. 4a, b), which is consistent with the previous reports in the literature^73,80–86^. Notably, the excitonic resonances on-cavity are sharper and exhibit significantly narrower linewidth than those off-cavity, reflecting the enhanced radiative decay in the confined cavity mode. This behavior is consistently observed across all the measured samples (see Supplementary Figs. S5, S6, and S7). It is important to note that the observation of multiple excitonic species in the literature typically requires very clean, hBN-encapsulated samples under gate-controlled doping. In contrast, our samples suffer from defects which lead to nonradiative broadening of the PL spectrum. As a result, some peaks only become distinct when cavity enhances radiative emission channels well above this background.

Importantly, we detect distinct trion peaks in the low-temperature spectra. Trions have been previously reported in gate-controlled experiments^84^ and occur only in n-doped WSe_2_. Their presence here indicates unintentional doping, likely introduced during the Au-assisted transfer process^92^. The unintentional doping density can be estimated from the measured exciton-trion energy separation. In the measured spectra, we extract ${E}_{{X}_{0}}=1.739{{\rm{eV}}}$ and ${E}_{{X}_{-}}=1.705{{\rm{eV}}}$ (Fig. 4e), corresponding to an exciton-trion splitting of Δ*E* ≈ 34 meV. Following the established relation between ΔE and the Fermi energy of the electron gas^84^, the trion binding energy sets the characteristic scale for dissociation into a free exciton and an electron. From the low-temperature measurements (Fig. 4), we extract the dissociation temperature of $60-70\,{{\rm{K}}}\,\left(\sim 5-6\,{\mathrm{meV}}\right)$. Together with the measured exciton-trion splitting, this implies the Fermi energy on the order of several tens of meV, corresponding to an electron density of ${{\rm{n}}}\sim {10}^{11}-{10}^{12}\,{{\mbox{cm}}}^{-2}$. In addition to trions, we also observe biexcitonic features, along with two additional low-energy peaks that are commonly attributed in the literature to defect-bound excitons^98^. In our measurements, these low-energy features persist even at room temperature, while trion and bi-exciton peaks vanish at elevated temperatures.

To investigate the behavior of these excitons, we performed power-dependent PL measurements at 5 K, both off and on the cavity (Fig. 4c and Fig. 4d, respectively). The spectra were analyzed using the Gaussian fits for the individual peaks, enabling us to quantitatively track their evolution with the excitation power (for fit comparisons, see Supplementary Fig. S8). An example fit is presented in Fig. 4e for an input power of *P*_in_ = 30 µW. The excitonic resonances undergo negligible spectral shifts even at the excitation powers exceeding 300 μW (Fig. 4c, d). At the same time, several peaks broaden markedly at higher powers and eventually merge with neighboring resonances. In contrast, the fitted peak amplitudes follow distinct power-law scaling behaviors (Fig. 4e): the bright and brightened excitons scale nearly linearly (${{\rm{\alpha }}}\approx 1$), biexcitons scale quadratically (${{\rm{\alpha }}}\approx 2$), and biexciton complexes exhibit intermediate scaling with the exponents in the range $\alpha \,\approx \,1$.

Since the low-energy bound excitonic modes persist up to room temperature, we performed energy-filtered lifetime measurements to investigate their role in the decay dynamics. Representative decay curves were collected for the on (green) and off (blue) cavity cases (Fig. 4g) utilizing the tunable femtosecond pulsed laser that was set to near-resonant excitation at the main exciton peak (730 nm). The PL was spectrally filtered using 10 nm bandpass filters, which were tilted as needed to selectively transmit bandwidths centered at 750, 760, and 780 nm (see Supplementary Fig. S9e for the measured filter responses at different angles). Clear differences in decay times are observed between the on- and off-cavity regions. At the same collection point, the variation of the detection wavelength—750, 760, and 780 nm—produces noticeable changes in the extracted dynamics. For reference, the decay times were also obtained using a 550 nm long-pass (LP) filter that collects the full PL response. These trends are summarized in Fig. 4h, where the fitted short decay components are plotted as a function of the detection wavelength. Interestingly, while the bound exciton emission centered at 780 nm would normally be expected to decay more slowly than the bright exciton emission at 750 nm, our data reveal an unexpected ∼2 times shorter decay time for the bound exciton compared to the bright excitons. As expected, the average decay times are also ∼ 2 times shorter in the cavity than in the off-cavity for the exciton emission at all wavelengths.

Finally, we measured the saturation behavior of the excitonic emission on and off the cavity. A clear difference is observed (Fig. 4i, j). On the cavity, the saturation curve rises more steeply and reaches saturation at 20 μW, whereas off-cavity the response is slower and saturates at higher powers (100 μW). Faster saturation observed on the cavity is consistent with the enhanced exciton-exciton interactions, further supporting that the field localization strengthens the interaction effects and can yield substantially larger excitonic nonlinearities. A detailed study of the resulting enhancement of two-photon nonlinearities is left for a follow-up work.

## Discussions

We showed that topology-optimized dielectric cavities provide an effective platform for controlling light–matter interactions in two-dimensional semiconductors. By harnessing adjoint-based inverse design, we realized CMOS-compatible silicon-on-sapphire (SoS) cavities with deeply subwavelength mode volumes and engineered far-field directivity, enabling deterministic coupling to excitons in monolayer WSe_2_. Photoluminescence mapping revealed raw photoluminescence enhancements approaching an order of magnitude relative to off-cavity, in quantitative agreement with simulations when fabrication tolerances are included. Importantly, the cavity coupling enables not only the amplified emission but also reshapes the excitonic dynamics. Specifically, the low-temperature spectroscopy showed sharpened excitonic resonances, including bright excitons, trions, and biexcitons, while the time-resolved measurements revealed cavity-mediated lifetime shortening and non-exponential decay signatures, which are indicative of the exciton-exciton interactions. These results provide direct evidence that the developed cavities accelerate radiative processes and mediate correlated exciton phenomena.

In the broader context of reported PL enhancements in 2D materials and related platforms (Table 1), our results are competitive with previously reported plasmonic structures. Reported enhancement values in the literature span several orders of magnitude, largely due to differing normalization conventions and geometric factors across experiments. To enable systematic comparison, Table I lists both the reported enhancement factors and raw values directly extracted from PL intensities or peak amplitudes, where available. We emphasize that the references included in the table were selected according to the following criteria: (1) the work reports photoluminescence (PL) emission enhancement, and (2) the enhancement is measured for WSe_2_ PL emission. Among the studies that satisfy these two criteria, we further down-selected to those reporting the largest enhancement factors to keep the table concise and focused. Studies lacking sufficient quantitative information for verification were excluded from this comparison.

**Table 1:** Comparison of photoluminescence (PL) and emission rate enhancements for WSe_2_ coupled to various photonic nanostructures

Year	Reference	Normalized enhancement	Raw enhancement	Geometry factor
2025	this work	10^3^	10–14	50–100
2025	Ref. ^33^	5	5	-
2025	Ref. ^6^	2.1 *×* 10^4^	14.23	1479.45
2024	Ref. ^7^	1.8 × 10^4^	7.44	2419.75
2023	Ref. ^8^	1.6 × 10^3^	1.1	1444
2023	Ref. ^9^	9 × 10^4^ (AS), 4 × 10^3^ (X_D_), 9 × 10^2^ (X_0_)	20 (AS), 12 (X_D_), 3 (X_0_)	50–430
2023	Ref. ^10^	6 × 10^3^ (X_IL_)	-	tip-enhanced
2022	Ref. ^11^	-	1.5	-
2018	Ref. ^12^	1.7 × 10^3^	7.5	225
2018	Ref. ^13^	0.7 × 10^3^	700	-
2018	Ref. ^37^	26	26	-
2018	Ref. ^21^	7	7	-
2018	Ref. ^14^	13(PL), 57(LT)	13(PL), 57(LT)	-
2017	Ref. ^15^	200	20	10
2016	Ref. ^16^	2.0 × 10^4^	1800	11.31
2015	Ref. ^29^	37 (LT)	-	-

This table summarizes reported values from high-performance literature specifically measuring WSe_2_ PL emission. “Raw enhancement” refers to the directly measured PL intensity ratio, while “normalized enhancement” accounts for the geometry factor (or area factor), which is defined as the area of the excitation beam spot divided by the effective area of the cavity hotspot. To enable a systematic comparison across different platforms, the normalized enhancement is reported as the product of the raw enhancement and this geometry factor

*AS* Anti-Stokes, *X*_*D*_ Dark excitons, *X*_0_ Bright exciton, *X*_*IL*_ Interlayer excitons, *PL* PL enhancement, *LT* lifetime shortening or Purcell factor

Overall, our findings establish topology-optimized dielectric cavities as a versatile, CMOS-compatible alternative to plasmonics for tailoring exciton-photon and exciton-exciton interactions. Unlike plasmonic architectures, these dielectric cavities achieve deep subwavelength confinement without ohmic loss, thereby preserving coherence while enabling efficient out-coupling. Beyond the enhanced PL, the combination of the strong near-field localization and controlled radiation offers a path toward unprecedented regimes of exciton nonlinearities, valley-selective emission, and cavity-enabled quantum many-body physics. Nevertheless, scalable fabrication of these structures presents significant fabrication challenges, including the proximity effects in EBL, shape distortions, and roughness due to reactive ion etching (RIE), which must be carefully addressed to preserve the designed optical performance. We anticipate that this platform will accelerate the integration of van der Waals materials into scalable quantum photonics, bridging the gap between fundamental exciton science and on-chip quantum technologies.

## Methods

### Adjoint topology optimization of metasurface cavities

We employ a gradient‑based topology‑optimization method^38,50,52^ in tandem with three‑dimensional finite‑difference time‑domain (FDTD) simulations to obtain silicon‑on‑sapphire cavity unit cells that confine optical modes deep below the diffraction limit. The design region is a single metasurface unit cell with periodicity ${{\rm{a}}}\approx 2{{{\rm{\lambda }}}}_{0}$ and *λ*_0_*/*(2*n*_Si_) thickness, which in our experiments ranges between 100 and 200 nm. The unit cell is bounded by PEC/PMC boundary conditions, which allows one to leverage the symmetric nature of the design and simulate only a quarter of the simulation domain. The design region is filled with a continuous material‑density field $\rho \,\left(x,\,y\right)\,\in \,\left[0,\,1\right]$, which is interpolated into the dielectric permittivity through

$$\varepsilon \left(x,y\right)={\varepsilon }_{{\mbox{air}}}\left[1-\rho \left(x,y\right)\right]+{\varepsilon }_{{\mbox{Si}}}\rho \left(x,y\right)$$

where ${{\rm{\rho }}}=0$ corresponds to air and ρ=1 to crystalline silicon. The figure of merit guiding the search is the electric‑field intensity at the central surface point

$${{\rm{F}}}={|{{\rm{E}}}\left(0,0,{z}_{{{\rm{surface}}}}\right)|}^{2}$$

whose topological gradient is obtained via the adjoint method. Writing objective function as ${{\rm{F}}}={\int }_{\chi }{{|E|}}^{2}{d}^{3}x$ over a small evaluation region χ, a first‑order perturbation of the permittivity gives

$${{\rm{\delta }}}F=2{{\mathrm{Re}}}{\int }_{x}E* \left(x\right)\delta E\left(x\right){d}^{3}x$$

Expressing δ*E* with the electric Green tensor and invoking reciprocity yields

$${{\rm{\delta }}}F=2{{\mathrm{Re}}}{\int }_{\psi }{{{\boldsymbol{P}}}}^{{{\rm{ind}}}}\left({x}^{\prime} \right)\cdot {{{\boldsymbol{E}}}}^{A}\left({x}^{\prime} \right){d}^{3}x^{\prime}$$

where ${{{\boldsymbol{P}}}}^{{{\rm{ind}}}}\left({x}^{{\prime} }\right)=\Delta {{\rm{\varepsilon }}}\left({x}^{{\prime} }\right){{{\boldsymbol{E}}}}^{{{\rm{fwd}}}}\left({x}^{{\prime} }\right)$ is the polarization density from the forward field and the adjoint field ${{{\boldsymbol{E}}}}^{A}$ is produced by a point electric dipole of complex amplitude $\frac{\partial F}{\partial {{\boldsymbol{E}}}}=2\,{{{\boldsymbol{E}}}}^{* }$ placed at the target location. Thus, each optimization step comprises a forward FDTD run to obtain ${{{\boldsymbol{E}}}}^{{{\rm{fwd}}}}$, an adjoint run driven by the dipole source to evaluate ${{{\boldsymbol{E}}}}^{A}$. Gradient scaling and update are performed using the adaptive moment estimation method (ADAM)^99^. The topology-optimization loop updates the continuous density field with adaptive moment gradient steps computed from adjoint sensitivities. At each iteration, the design variable ρ is filtered by convolution with a Gaussian kernel in Fourier space with 20 nm radius to suppress features below the minimum fabricable size. The filtered design field then undergoes a smoothed Heaviside projection whose sharpness parameter *β* is gradually ramped from 1 to 1000, systematically pushing ρ toward binary values and enforcing manufacturability. We iterate this sequence-forward solve, adjoint solve, filtered ADAM update, and binarization for roughly 200–250 iterations, based on FOM saturation and design field binarization.

### Fabrication

A 100 nm thick epitaxial SoS wafer is diced into 6.5 by 6.5 mm pieces. The pieces are then cleaned by ultrasonication in acetone, isopropanol, and deionized water, and dried with dry N_2_. The sample surface is dehydrated by heating in a nitrogen atmosphere and primed with hexamethyldisilazane (HMDS) vapor in a Yield Engineering Services YES LP-III vacuum oven.

The sample is spin-coated with a 50 nm film of electron beam resist (ZEP 520A) diluted 1:2 in anisole at 4000 rpm for 45 s, and the coated sample is baked on a hotplate at 180 °C for 3 min. 100 by 100 µm^2^ meta-cavity arrays are written in the electron beam resist using an Elionix ELS-BODEN 150 electron beam lithography system with a 150 keV electron beam at a current of 200 pA and base exposure dose of 325 µC*/*cm^2^. Multiple copies of the array are written with various combinations of unit cell scaling (detuning) and proximity effect correction (PEC) parameters. The sample is developed in chilled (approx. 3 °C) o-xylene for 30 s, rinsed in isopropanol for 30 s, and dried with dry N_2_.

The developed pattern is transferred to the underlying silicon by inductively coupled reactive ion etching (ICP-RIE) in an SPTS Omega LPX Rapier reactive ion etcher. After etching, the remaining electron beam resist is removed by rinsing in acetone and isopropanol, followed by 4 min of O_2_ plasma etching in a Matrix Integrated Systems 105 plasma asher.

### Flake transfer

Monolayer WSe_2_ flakes were prepared using a gold-template-assisted mechanical exfoliation technique adapted from Wu et al.^92^. Bulk WSe_2_ crystals were exfoliated onto a patterned Au grid on a Si/SiO_2_ substrate immediately after broad Ar ion beam polishing. Broad ion beam polishing was performed using a JEOL IB-19500CP Cross Section Polisher with the beam parallel to the substrate surface. The procedure recovers the Au surface and enables enhanced exfoliation yield and uniformity similar to those of freshly deposited Au thin films. Monolayer regions were identified via optical contrast and subsequently transferred along with an h-BN capping thin flake onto our patterned Si substrate using a dry transfer method with a polycarbonate (PC)/polydimethylsiloxane (PDMS) stamp. After the transfer, the samples were soaked in chloroform to remove residual PC and improve adhesion. Monolayer thickness was confirmed by photoluminescence spectroscopy and Raman spectroscopy. Original samples in the experiment that had flake thickness of a few to tens of nanometers were mechanically exfoliated from bulk crystals using a standard adhesive tape method and their thickness was measured with atomic force microscopy (AFM). These thicker flakes were also transferred onto the patterned Si substrate using a dry transfer method with a polycarbonate (PC)/PDMS stamp.

### Optical measurements

All optical measurements were performed using custom-built scanning confocal microscopy setups. Room-temperature characterization was conducted on an inverted confocal microscope platform, while low-temperature measurements used a top-access closed-loop helium cryostat equipped with a vacuum-compatible, high NA objective. For the room temperature setup, sample scanning was achieved using a nanopositioning stage. For the low-temperature setup, a 4F scanning technique using dual-axis scanning galvo system was utilized. The excitation and PL collection for both setups were configured in a reflection geometry. A detailed schematic and component list for each setup can be found in Supplementary Figs. S1 and S10.

### Continuous-wave PL, spectral analysis, and saturation measurements

For continuous-wave (CW) experiments, samples were excited with a 532 nm diode laser. The collected PL was passed through a set of long-pass and band-pass filters to isolate the emission band centered at 750 nm. The signal was then spatially filtered with a confocal pinhole and detected by a single-photon avalanche diode (SPAD). Saturation curves were obtained by measuring the steady-state PL intensity as a function of excitation power. For spectral analysis, the PL was directed to a visible-to-near-infrared spectrometer, and all spectra were corrected for detector response and background.

### Time-resolved PL measurements

Time-resolved PL (TRPL) measurements were performed using a tunable femtosecond Ti:Sapphire laser for both above-gap (520 nm, via second harmonic generation) and near-resonant (730 nm) excitation. The fluorescence decay traces were recorded using a high-resolution SPAD and a time-correlated single-photon counting (TCSPC) module.

### Data acquisition and analysis

All measurements were controlled and synchronized using custom LabVIEW software. The resulting fluorescence decay curves were analyzed using nonlinear least-squares fitting routines in Julia and Python. To accurately model the observed dynamics, the data were fit to several models, including single exponential, biexponential, and stretched exponential forms, after deconvolution with the IRF. The full mathematical descriptions of the models and the fitting procedure are detailed in S5.

## Supplementary information

Topology-Optimized Dielectric Cavities for Enhanced Excitonic Light Emission from WSe<sub>2</sub> SUPPLEMENTARY INFORMATION

## Data Availability

The data that support the findings of this study have been included in the main text and Supplementary Information. All other relevant data supporting the findings of this study are available from the corresponding authors upon request. The codes used for the lifetime and spectra fitting are available from the corresponding authors upon reasonable request.
