# Supplementary material for: Topology-optimized dielectric cavities for enhanced excitonic light emission from WSe2
Source: Light Sci Appl. 2026 Oct 13;15:387. doi: 10.1038/s41377-026-02436-w (PMC13658778; doi:10.1038/s41377-026-02436-w)
Supplement: Supplementary file 1 [file 41377_2026_2436_MOESM1_ESM.pdf]

Topology-Optimized Dielectric Cavities for Enhanced Excitonic Light Emission from WSe_2

– SUPPLEMENTARY INFORMATION –

Owen Matthiessen,[1,2,*] Brandon Triplett,[1,2,*] Omer Yesilyurt,[1,2,*] Davide Cassara,[3,*] Karthik Pagadala,[1,2] Morris M. Yang,[1,2] Andres E. Llacsahuanga Allcca,[4] Hamza Ather,[4,1,2] Colton Fruhling,[1,2] Abhishek Bharatbhai Solanki,[1,2] Yong P. Chen,[1,5,2,4] Hadiseh Alaeian,[1,5,2,4] Alexander V. Kildishev,[1,2] Vladimir M. Shalaev,[1,5,2,4,†] Federico Capasso,[3,‡] Alexandra Boltasseva,[1,5,2,§] and Vahagn Mkhitaryan[1,2,¶]

[1] *Elmore Family School of Electrical and Computer Engineering, Purdue University, West Lafayette, IN 47906, USA*
[2] *Birck Nanotechnology Center, Purdue University, West Lafayette, IN 47906, USA*
[3] *John A. Paulson School of Engineering and Applied Sciences, Harvard University, Cambridge, MA 02138, USA*
[4] *Department of Physics and Astronomy, Purdue University, West Lafayette, IN 47907, USA*
[5] *Purdue Quantum Science and Engineering Institute, Purdue University, West Lafayette, IN 47907, USA*

Contents

S1. Low-temperature optical setup 2

S2. Raman Spectroscopy 3

S3. Atomic Force Microscopy 4

S4. Fabrication results 4

S5. Lifetime 5
- S5.1 Measurement 5
- S5.2 Fitting 5
- S5.3 Supplemental data 6
- S5.4 Spatially Resolved Lifetime Mapping 7
- S5.5 Model Validation via Reduced Chi-Squared Analysis 7
- S5.6 Power dependence of effective decay time 9

S6. Low Temperature Spectra 9
- S6.1 Power and temperature dependence of PL spectra 9

S7. Room Temperature Optical Setup 13

S8. Extraction of Doping Density 13

S9. Normalized Enhancement Calculation 14

References 14

[*]These authors contributed equally.
[†]Corresponding author: shalaev@purdue.edu
[‡]Corresponding author: capasso@seas.harvard.edu
[§]Corresponding author: aeb@purdue.edu
[¶]Corresponding author: vmkhit@gmail.com

S1. LOW-TEMPERATURE OPTICAL SETUP

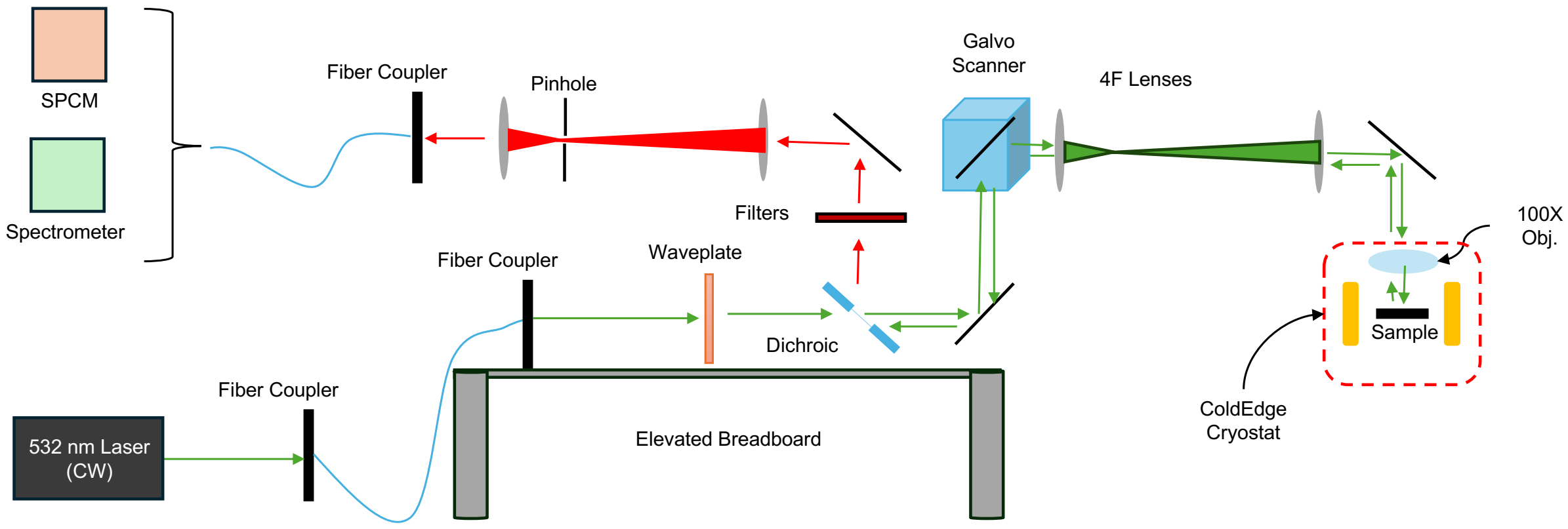

FIG. S1: **Schematic of low-temperature optical setup.** A 532 nm CW laser (Cobolt) is focused onto the sample inside a closed-loop helium cryostat via a high-NA objective (Attocube). Scanning is performed with a 2D galvo system. The collected PL is spatially filtered with a 50 µm pinhole and sent to one of two detection arms: a SPAD (Excelitas) for photon counting or a spectrometer (Princeton Instruments) with a liquid-nitrogen-cooled CCD for acquiring emission spectra.

Low-temperature PL measurements were performed using a custom-built cryogenic confocal microscope (Fig. S1). The setup features a ColdEdge Stinger Hydra cryostat with a closed-loop liquid helium cooling system. Excitation and collection were achieved with a low-temperature compatible objective (Attocube, LT-APO/532-RAMAN/0.82 NA) mounted inside the cryostat. Sample scanning was performed using a 2D large-beam galvo system (Thorlabs, GVS012).

The excitation source was a fiber-coupled 532 nm CW laser (Cobolt 08-DPL, Hubner Photonics). Collected PL was spatially filtered with a 50 µm pinhole before detection. The signal was directed either to a SPAD (Excelitas, SPCM-AQRH) for photon counting or to a Princeton Instruments HRS-300 spectrometer for spectral characterization. The spectrometer was equipped with interchangeable diffraction gratings (1200 g/mm and 600 g/mm) and a liquid-nitrogen-cooled PyLoN CCD camera for low-noise spectral acquisition.

S2. RAMAN SPECTROSCOPY

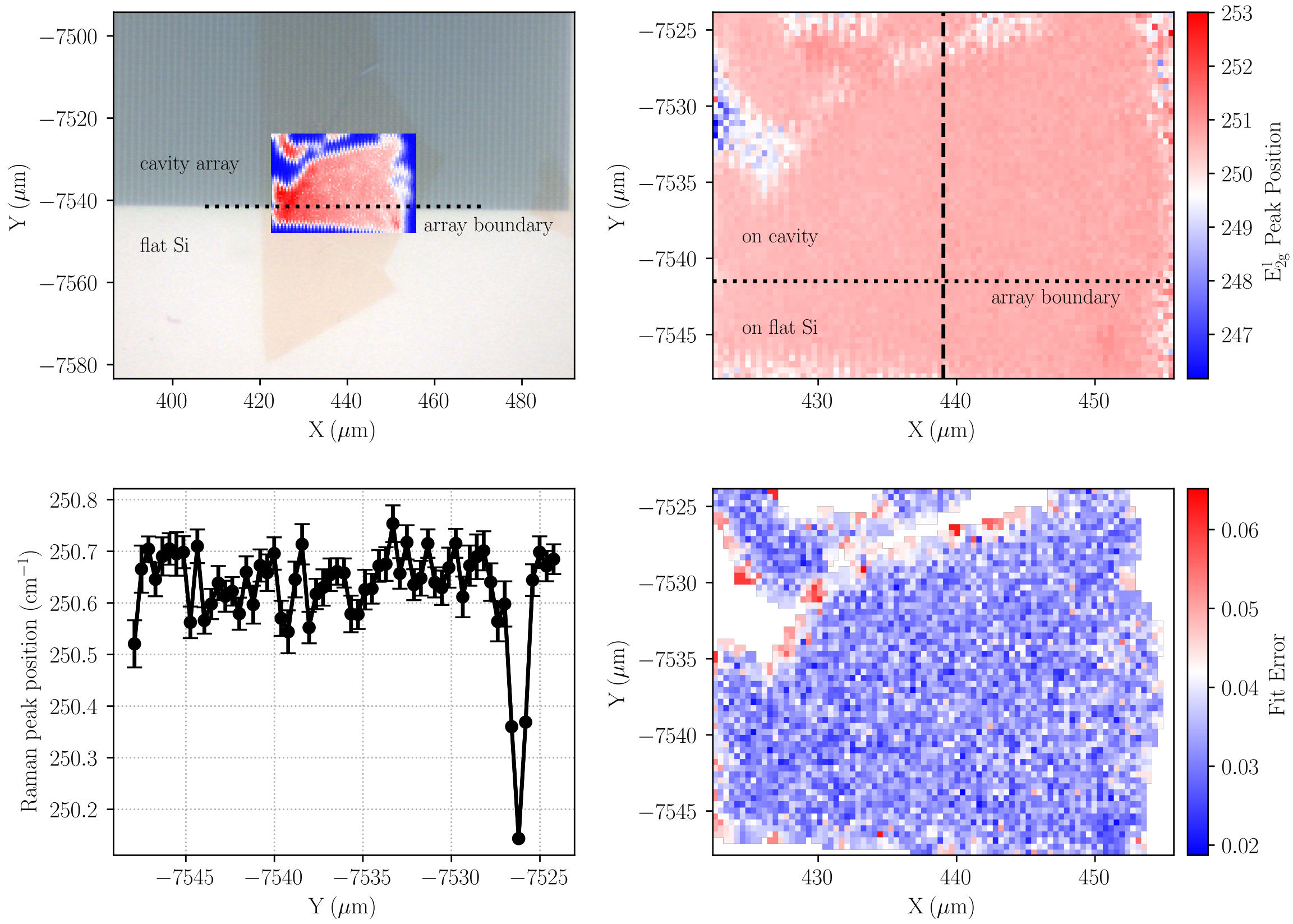

FIG. S2: **Raman mapping of a WSe_2 flake.** **a**,**b**, Map of the Raman E^1_{2g} peak position superimposed on an optical microscope image. **c**, A line profile of the peak position across the flake, indicating strain variation. **d**, Fit errors from the analysis.

Raman maps were acquired using a Thermo Scientific DXR3xi Raman imaging microscope with a 532 nm excitation laser and a spatial resolution of 0.4 μm per pixel. Point spectra from the maps were fit using a dual Lorentzian oscillator model to extract the peak positions of the E^1_{2g} and A_{2g} modes of WSe_2. The peak positions were extracted from the resulting fits and the map of the E^1_{2g} peak position superimposed on an optical microscope image is shown in Figure S2a. S2b shows the Raman E^1_{2g} Peak position map, highlighting the areas where the WSe_2 flake is on-cavity and off-cavity. A dashed-line profile of peak positions is displayed in S3b and plotted in S2c. The fit errors shown in S2d clearly indicate the WSe_2 flake compared to the background.

S3. ATOMIC FORCE MICROSCOPY

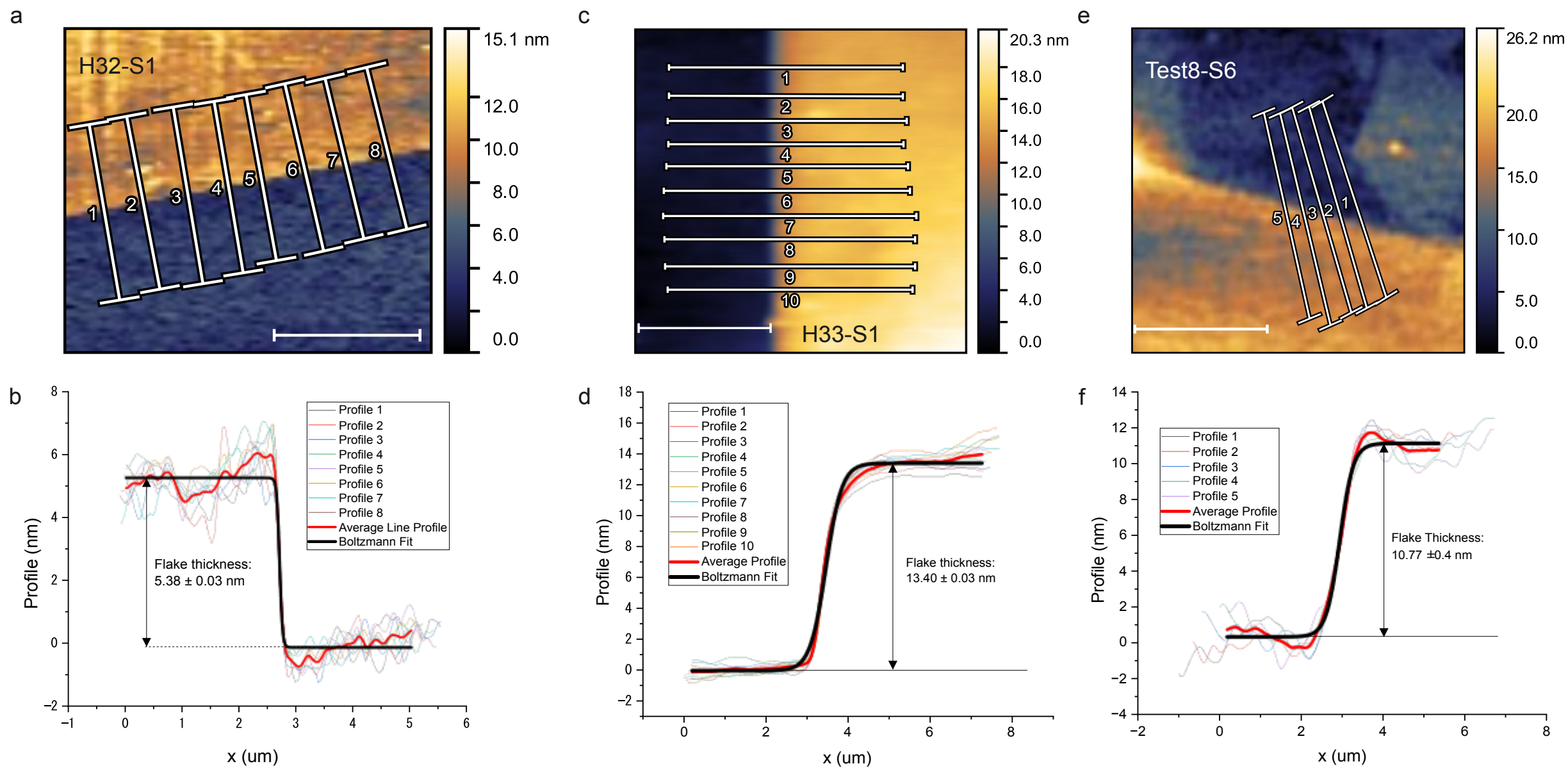

FIG. S3: **hBN thicknesses in the different samples as estimated using atomic force microscopy a**,**c**,**e**, AFM height maps of samples labeled H32-S1, H33-S1, and Test8-S6, respectively. White lines indicate the locations of individual height profiles used to determine the flake thickness. Scale bars are 5 µm. **b**,**d**,**f**, Height profiles along the lines shown in (**a**,**c**,**e**), with individual profiles, the average profile in red, and a Boltzmann fit to the average profile in black. The extracted flake thicknesses from the Boltzmann fits are indicated in each plot.

AFM scans were performed on 3 samples using a Park Systems AFM in NCFM mode. Area scans were saved and processed with Gwyddion software for surface leveling and line-scan fitting.

S4. FABRICATION RESULTS

The small, dense features and high fidelity demanded by the inverse design process impose unique constraints on fabrication. The etching procedure, in particular, has to be carefully optimized. Small features require a thin etch mask, which in turn requires high etch selectivity; narrow gaps require straight sidewalls; and high sensitivity to fabrication errors requires smooth sidewalls. We opted for a non-switched Bosch-type etch using SF_6, C_4F_8, and CHF_3 for simultaneous etching and sidewall passivation. The operating pressure, gas flows, ICP power, and bias power were optimized to balance sidewall passivation and horizontal facet fluoropolymer removal, yielding an etch with a selectivity of approximately 5:1 against the etch mask and smooth sidewalls at an angle of 88 degrees. A meta-cavity array produced using the final optimized etch can be seen in S4a. Etching results produced by intermediate optimization steps and their respective etching parameters are shown in S4b-c.

A key technique used as part of the previously described optimization was automated contrast-based segmentation and contour detection. Secondary electron imaging is generally used to capture topography on the scale of the features of our meta-cavities. As a result, sharp corners, such as those at the edges of etched regions, exhibit a so-called "secondary electron glow." We developed a script utilizing thresholding and denoising algorithms to isolate the secondary electron glow present in our SEM images, thereby extracting the contours of the as-fabricated meta-cavities. Doing so enables a direct comparison with the blueprint design, reduces the optimization cycle time, provides valuable insights into the impact of processing variations on the final fabricated structure, and enables simulation of the as-fabricated structure. Examples of SEM images and the detected contours, along with comparisons of the detected contours to the blueprint designs, are shown in S4d-g.

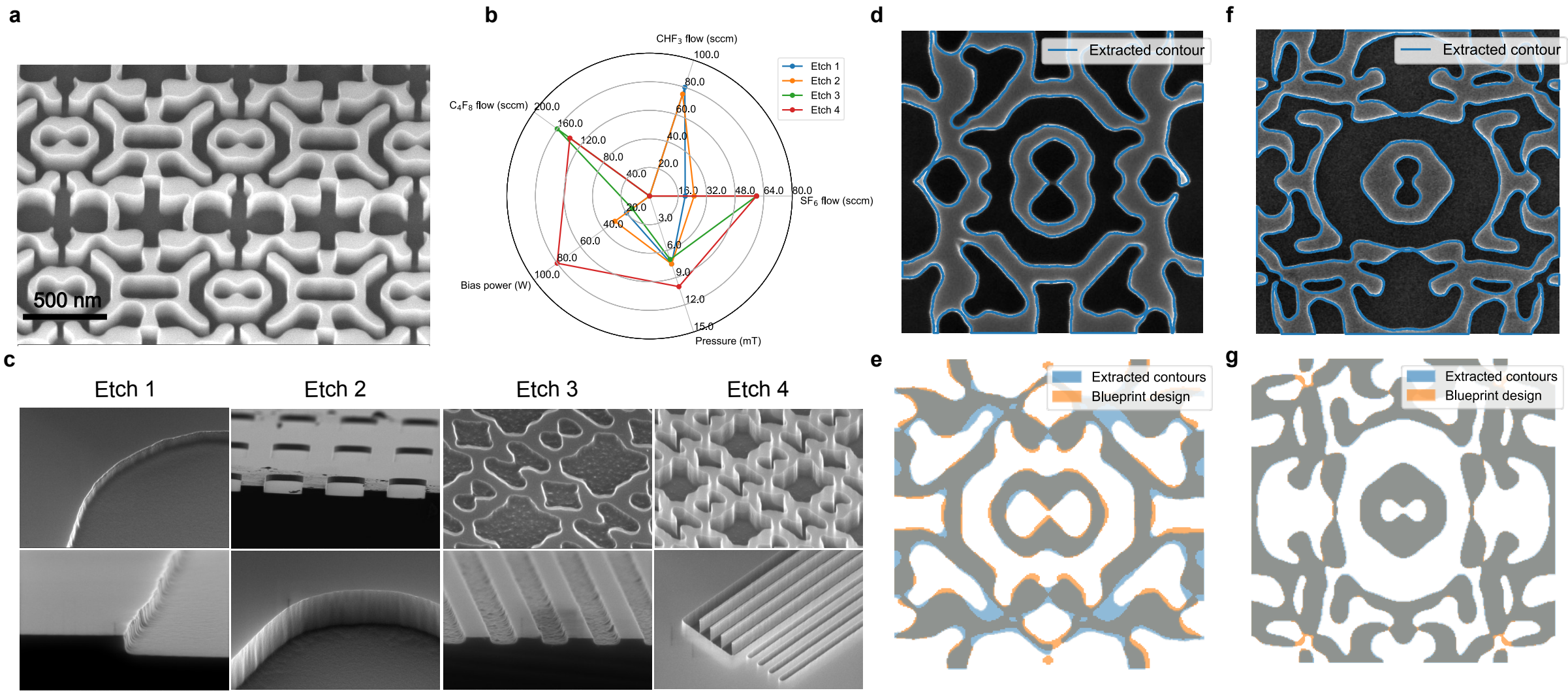

FIG. S4: **Etch Optimization and cavity shape distortions compared to the original design**. **a**, representative SEM image of etched cavity. **b**, parameters of four selected etches from the optimization process. **c**, representative SEM images of four selected etches from the optimization process. **d**,**f**, SEM images of the fabricated structure and detected contours. **e**,**g**, comparison of fabricated structure and blueprint design.

S5. LIFETIME

S5.1 Measurement

The lifetime measurements were conducted using the room-temperature confocal microscope equipped with the femtosecond Ti:Sapphire laser and time-correlated single-photon counting (TCSPC) module as shown in Fig. S5. The laser was operated at 1040 nm and frequency doubled to 520 nm. The excitation powers ranged from 1-50 μW. The collection time was set to 10 seconds. The instrument response function (IRF) of the system was measured separately by detecting scattered laser light from a metal post. Energy-filtered measurements were performed using $\pm$10 nm bandpass filters centered at 750 and 760 nm. To access 780 nm, an 800 nm bandpass filter was tilted by 25°. The transmission spectra of the filters are shown in Fig. S9e. For measurements with the tilted 800 nm filter, the collection time was doubled from 10 to 20 s to compensate for reduced efficiency.

S5.2 Fitting

The photoluminescence lifetime data were fit to several candidate models to identify the most accurate physical description of the decay process. The fitting was performed in Python using the `lmfit` package [1]. We used a convolution-based fitting technique in which each theoretical model was convolved with the measured instrument response function (IRF) before comparison with the experimental data. This approach is numerically more stable than direct deconvolution, especially for noisy signals.

The model parameters, such as the fast and slow lifetimes (τ_1, τ_2) and the stretching factor (β), were optimized using a weighted least-squares minimization routine. The fits were weighted by the Poisson noise of the photon counts ($\frac{1}{\sqrt{\text{counts}}}$) and used a strict convergence tolerance of 10^{-8}. We tested three candidate models: (1) a multi-exponential, (2) a stretched exponential, and (3) a mixed model. The mixed model was ultimately selected for presentation in the main paper because it consistently yielded the best fits, as determined by reduced chi-squared values close to unity ($\chi^2_{\text{red}} \approx 1$) and a visual inspection of the residuals. Fits with poor statistics or systematic residuals were rejected from the final analysis. The model functions are listed below.

Stretched exponential model:

$$I(t) = A \exp\left[-\left(\frac{t}{\tau}\right)^{\beta}\right]$$

Multi-exponential model ($n = 3$):

$$I(t) = A_1 e^{-t/\tau_1} + A_2 e^{-t/\tau_2} + A_3 e^{-t/\tau_3}$$

Mixed model:

$$I(t) = A_1 \exp\left[-\left(\frac{t}{\tau_1}\right)^{\beta}\right] + A_2 e^{-t/\tau_2}$$

IRF model:

$$I(t) = (y_x * y_g)(t) + y_0, \tag{S1}$$

$$y_x(t) = A\left[1 + \tanh\left(\frac{t-\mu}{t_r}\right)\right]\left(e^{-\frac{t-\mu}{t_d}} + Be^{-t/\tau}\right), \tag{S2}$$

$$y_g(t) = \frac{1}{\sigma\sqrt{2\pi}} \exp\left[-\frac{(t-\mu)^2}{2\sigma^2}\right]. \tag{S3}$$

S5.3 Supplemental data

The analogous figures for our other extensively measured samples (H33-S1 and Test-8 S6), corresponding to Figure 4 in the main text (H32-S1), are shown below.

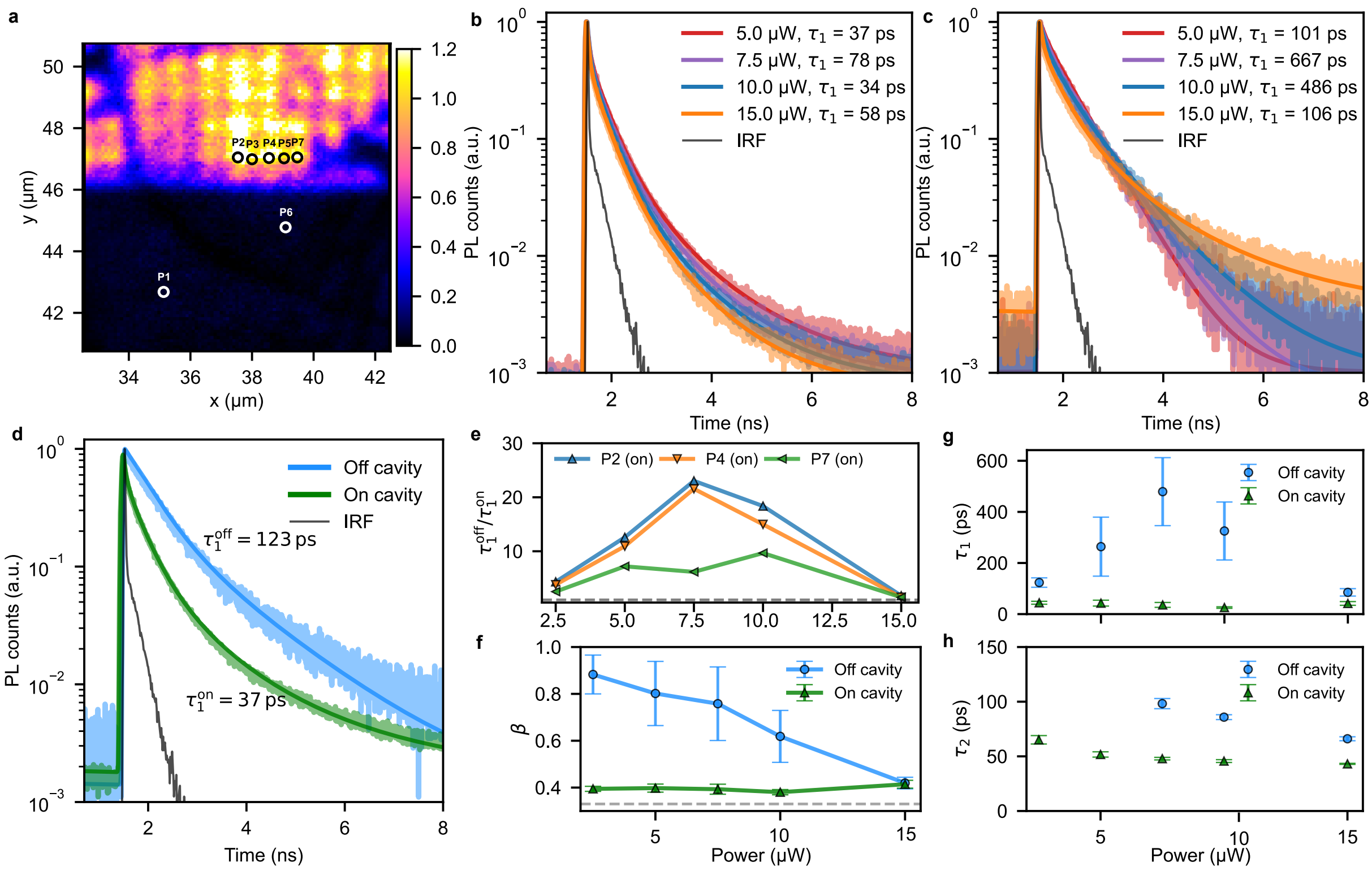

FIG. S5: **Lifetime fitting of non-exponential decay with mixed modeling for sample Test-8 S6.** **a**, PL map showing selected measurement points on and off the cavity. **b**, Decay curves from a cavity location (Point 2) at varying input powers, with corresponding mixed-model fits. The gray trace shows the instrument response function (IRF). **c**, Decay curves from a reference location off the cavity (Point 1), with corresponding fits and IRF as in (**b**). **d**, Averaged decay curves and fits on and off the cavity. **e**, Ratio of short decay times $\tau_1^{\mathrm{off}}/\tau_1^{\mathrm{on}}$ for selected points as a function of pump power. **f**, Stretching factor β as a function of pump power on and off the cavity.**g**, Averaged short decay time τ_1 on and off the cavity as a function of pump power, showing a nonlinear decrease off-cavity and a nearly linear dependence on-cavity. **h**, Same as (**g**), but for the long decay time τ_2.

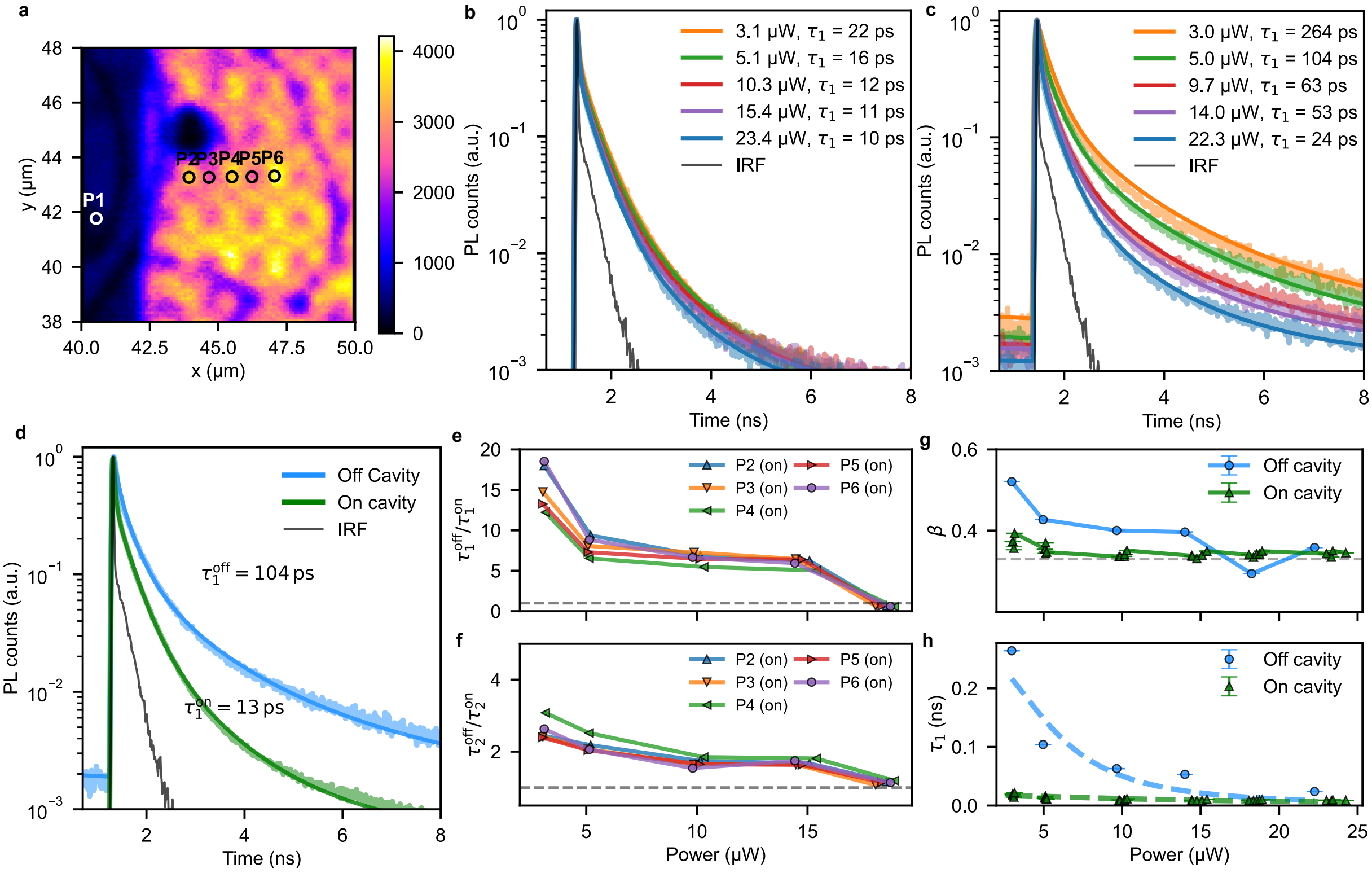

FIG. S6: **Lifetime fitting of non-exponential decay with mixed modeling for sample H33-S1.** **a**, PL map showing selected measurement points on and off the cavity. **b**, Decay curves from a cavity location (Point 6) at varying input powers, with corresponding mixed-model fits. The gray trace shows the instrument response function (IRF). **c**, Decay curves from a reference location off the cavity (Point 1), with corresponding fits and IRF as in (**b**). **d**, Averaged decay curves and fits on and off the cavity. **e**, Ratio of short decay times $\tau_1^{\text{off}}/\tau_1^{\text{on}}$ for selected points as a function of pump power. **f**, Same as (**e**), but for the long decay time τ_2 from the mixed-model fits. **g**, Stretching factor β as a function of pump power on and off the cavity. **h**, Averaged short decay time τ_1 on and off the cavity as a function of pump power.

While multiple devices were characterized, the data from sample H32-S1 is highlighted in the main text. This sample provided the most comprehensive dataset for the off-cavity regions, allowing for the most reliable comparison between cavity-coupled and uncoupled emission dynamics. is We include the analogous data for two other representative samples (H33-S1 and Test 8 S6) in Supplementary Figs. S5 and S6. Although the off-cavity measurements for these samples contained fewer data points or exhibited larger fit uncertainties due to lower signal, the on-cavity decay dynamics were in excellent agreement across all measured devices, thereby confirming the generality of our conclusions.

S5.4 Spatially Resolved Lifetime Mapping

We performed spatially resolved lifetime measurements to map the variation of decay parameters across the sample surface. These maps were acquired by scanning the sample in 1-μm steps and recording a full photoluminescence decay curve at each pixel. Each decay curve was then fit using the mixed model described previously. The extracted fitting parameters (e.g., τ_1, τ_2, β) were then plotted as a function of position, as shown in Supplementary Fig. S9a,b, and c. The spatial resolution of these maps is likely limited by mechanical drift over the prolonged (multi-hour) acquisition period.

S5.5 Model Validation via Reduced Chi-Squared Analysis

To quantitatively validate our choice of fitting model, we compared the reduced chi-squared (χ^2_{red}) values for all three candidate models across our datasets, as shown in Supplementary Fig. S7. A fit was considered reliable and included in our statistical analysis only if it met the criterion of $\chi^2_{\text{red}} < 2$.

Fits that failed this criterion were predominantly from low-power measurements with a poor signal-to-noise ratio and were consequently excluded. The results clearly demonstrate that both the mixed and multi-exponential models consistently outperform the single stretched-exponential model, providing strong justification for our use of the mixed model in the main analysis.

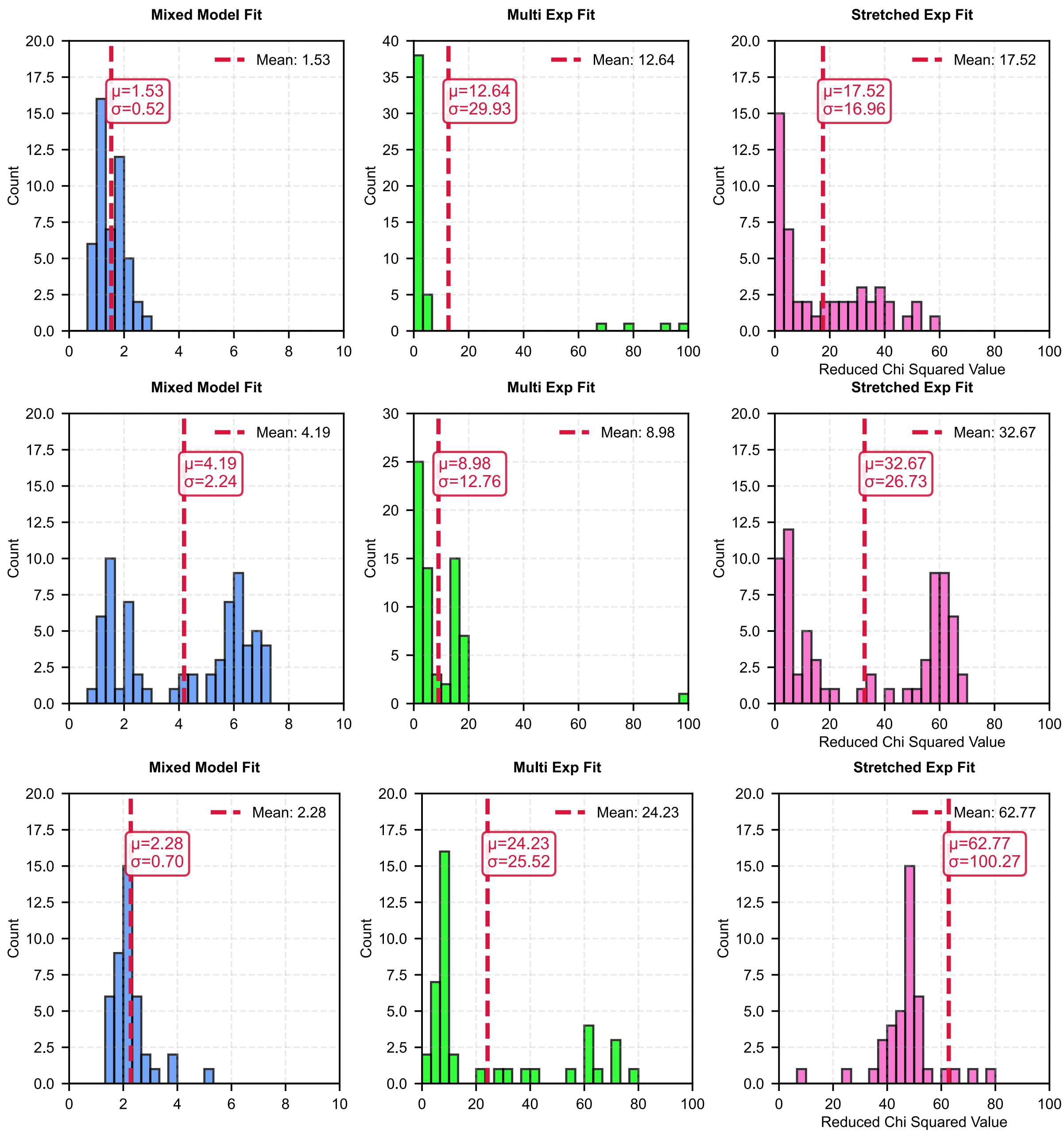

FIG. S7: **Comparison of reduced chi-squared values for sample Test-8 S6, H32-S1, and H33-S1.** Histograms show the distribution of the reduced chi-squared (χ^2_{red}) values obtained from fitting the lifetime decay data with three different models: the mixed model (left), the multi-exponential model (center), and the stretched exponential model (right). The distribution for the mixed model is tightly centered near unity ($\mu = 1.53$), indicating a consistently robust fit. In contrast, the other models yield significantly larger average χ^2_{red} values and broader distributions.

S5.6 Power dependence of effective decay time

The dynamics of the carrier density n can be described by the continuity equation [2]:

$$\frac{\partial n}{\partial t} = D\nabla^2 n - \left(\frac{1}{\tau_{\mathrm{sp}}}n + Bn^2 + Cn^3\right) \tag{S4}$$

where $D\nabla^2 n$ on the right-hand side denotes the spatial carrier diffusion, and the remaining, subtracted part includes linear non-radiative ($\tau_{\mathrm{sp}}^{-1}n$), radiative (Bn^2), and the Auger recombination (Cn^3) terms.

One can now assume the decay dynamics can be approximated with an effective decay time, and write

$$n(t) = n_0 e^{-t/\tau_{\mathrm{eff}}} \tag{S5}$$

One can find the effective decay time as

$$\tau_{\mathrm{eff}} = -\frac{n_0}{\mathrm{d}n(t)/\mathrm{d}t|_{t=0}} \tag{S6}$$

Using this and the equation S4, and ignoring the contribution from the diffusion term, we can now express the effective decay time as

$$\tau_{\mathrm{eff}} = \frac{\tau_{\mathrm{sp}}}{1 + \tau_{\mathrm{sp}}Bn_0 + \tau_{\mathrm{sp}}Cn_0^2} \tag{S7}$$

Now, since the initial exciton density is proportional to input power $n_0 = GP$ at low power limit, we can get the effective power dependence for the observed decay time

$$\tau_{\mathrm{eff}} = \frac{\tau_{\mathrm{sp}}}{1 + \tau_{\mathrm{sp}}BGP + \tau_{\mathrm{sp}}G^2CP^2} \tag{S8}$$

S6. LOW TEMPERATURE SPECTRA

Emission peaks for WSe_2 are fitted via a Julia curve fitting function with Gaussian and Voigt distributions with propagating error statistics. Residuals are plotted as the difference between raw spectra data and fitted curve amplitudes. Eight peaks between 1.55 eV and 1.74 eV were identified.

S6.1 Power and temperature dependence of PL spectra

Power-dependent spectra measured at 5 K from 30 μW to 360 μW. Temperature dependent data measured at 55 μW input power from 5 K up to 100 K. Peak amplitudes of each identified Gaussian and Voigt peaks and peak energies were plotted and fitted against the power law to match the distribution identifying as exciton, bi-exciton, or trion through coefficient α.

Lorentz model [3]: The causal Lorentz model for emission/absorption spectra is,

$$f_{\mathrm{L}}(\omega; A_{\mathrm{L}}, \omega_0, \gamma) = A_{\mathrm{L}} \frac{\gamma\omega}{(\omega_0^2 - \omega^2)^2 + 4\gamma^2\omega^2}$$

Decomposing the Lorentzian into single poles with $\omega_0^2 = \Omega_{\mathrm{L}}^2 + \gamma^2$ gives [4],

$$f_{\mathrm{L}}(\omega; A_{\mathrm{L}}, \omega_0, \gamma) = f_{\mathrm{L}}(\omega; A_{\mathrm{L}}, \Omega_{\mathrm{L}}, \gamma) = \frac{A_{\mathrm{L}}}{2\Omega_{\mathrm{L}}}\left[\frac{\gamma}{(\omega - \Omega_{\mathrm{L}})^2 + \gamma^2} - \frac{\gamma}{(\omega + \Omega_{\mathrm{L}})^2 + \gamma^2}\right],$$

It is a common practice to ignore the second (blue-marked) part in spectroscopic fits and use a non-causal, single-term version,

$$f_{\mathrm{L}}(\omega; A_{\mathrm{L}}, \Omega_{\mathrm{L}}, \gamma) = A_{\mathrm{L}} \frac{\gamma}{\gamma^2 + (\omega - \Omega_{\mathrm{L}})^2}$$

that also matches the scaled Cauchy-Lorentz probability density function (PDF).

Causal Gaussian model (see e.g., [4–6]):

$$f_{\mathrm{G}}(\omega; A_{\mathrm{G}}, \Omega_{\mathrm{G}}, \sigma) = \frac{A_{\mathrm{G}}\sqrt{\pi}}{2\sigma\sqrt{2}}\left[\mathrm{e}^{-\frac{(\omega-\Omega_{\mathrm{G}})^2}{2\sigma^2}} - \mathrm{e}^{-\frac{(\omega+\Omega_{\mathrm{G}})^2}{2\sigma^2}}\right]$$

We can also ignore the second (orange) part in the spectroscopic fits and use a non-causal, single-term version,

$$f_{\mathrm{G}}(\omega; A_{\mathrm{G}}, \Omega_{\mathrm{G}}, \sigma) = \frac{A_{\mathrm{G}}\sqrt{\pi}}{2\sigma\sqrt{2}}\mathrm{e}^{-\frac{(\omega-\Omega_{\mathrm{G}})^2}{2\sigma^2}}$$

which is a scaled Gaussian PDF.

Causal Voigt (Gauss-Lorentz) model [4, 7]:

$$f_{\mathrm{V}}(\omega; A_{\mathrm{V}}, \Omega_{\mathrm{V}}, \gamma) = \frac{A_{\mathrm{V}}\sqrt{\pi}}{2\sigma\Omega_{\mathrm{V}}\sqrt{2}}\Re\left[w\left(\frac{\omega+\imath\gamma-\Omega_{\mathrm{V}}}{\sigma\sqrt{2}}\right) - w\left(\frac{\omega+\imath\gamma+\Omega_{\mathrm{V}}}{\sigma\sqrt{2}}\right)\right]$$

Ignoring the last (purple) term yields a simpler, single-term version,

$$f_{\mathrm{V}}(\omega; A_{\mathrm{V}}, \Omega_{\mathrm{V}}, \gamma) = \frac{A_{\mathrm{V}}\sqrt{\pi}}{2\sigma\Omega_{\mathrm{V}}\sqrt{2}}\Re\left[w\left(\frac{\omega+\imath\gamma-\Omega_{\mathrm{V}}}{\sigma\sqrt{2}}\right)\right]$$

This simplified, single-term dispersion model $f_{\mathrm{V}}(\omega; ...)$ is obtained as the convolution of the single-term Lorentzian and Gaussian functions $f_{\mathrm{V}}(\omega) = (f_{\mathrm{L}} * f_{\mathrm{G}})(\omega)$; $w(z)$ denotes the Faddeeva (Kramp) function of a complex argument

$$z = \frac{\omega - \Omega_V + i\gamma}{\sigma\sqrt{2\pi}},$$

which apparently inherits all the critical parameters, i.e., offset $\Omega_{\mathrm{V}} = \Omega_{\mathrm{G}} + \Omega_{\mathrm{L}}$ and broadening (γ and σ), from both convolved functions. The Faddeeva function [8] is related to the scaled complex complementary error function as

$$w(z) = \exp(-z^2)\mathrm{erfc}(-iz) = \mathrm{erfcx}(-iz) = \exp(-z^2)\left[1 + \frac{2i}{\sqrt{\pi}}\int_0^z \mathrm{e}^{t^2}\mathrm{d}t\right]$$

An open-source C++ code for approximating the Faddeeva function (with wrappers for C, Matlab, GNU Octave, Python, R, Scilab, and Julia) was written by Steven Johnson [9]. Very efficient, few-term approximants for $w(z)$ are also available in [4, 6].

Fitting with the Single-Term Gauss and Voigt functions: The fitted $\Omega_{\mathrm{G,V}}$ are defined as the peak energy for the eight peaks that correspond to the Raman spectra. Fitting errors are defined as the differences between the fitted and raw spectra. The subsequent fitted parameters are fitted again into a power law. Both the Voigt and Gaussian models fit the curves well, with the same number of peaks across the range of input powers. Several local maxima in the error arise from the negative counts in the spectrometer noise. Several other fitting uncertainties arise from the interplay among overlapping peaks, which are mitigated by setting the fit tolerance in the code. Energies of each peak remain consistent in power-dependent spectra, with negligible changes arising from fitting errors. Peak energies blue shifts with increasing temperature, albeit with large fitting uncertainties and poor mapping of each peak's change as a function of temperature due to some peak disappearing at higher temperatures.

Power law:

$$I(P_{\mathrm{in}}) = AP_{\mathrm{in}}^{\alpha}$$

The fitted Gaussian and Voigt peaks in temperature-dependent data were further fitted as part of the Varshni equation to obtain the binding energies. The binding energies and the amount of sample doping from the Au-assisted transfer process can be further extracted.

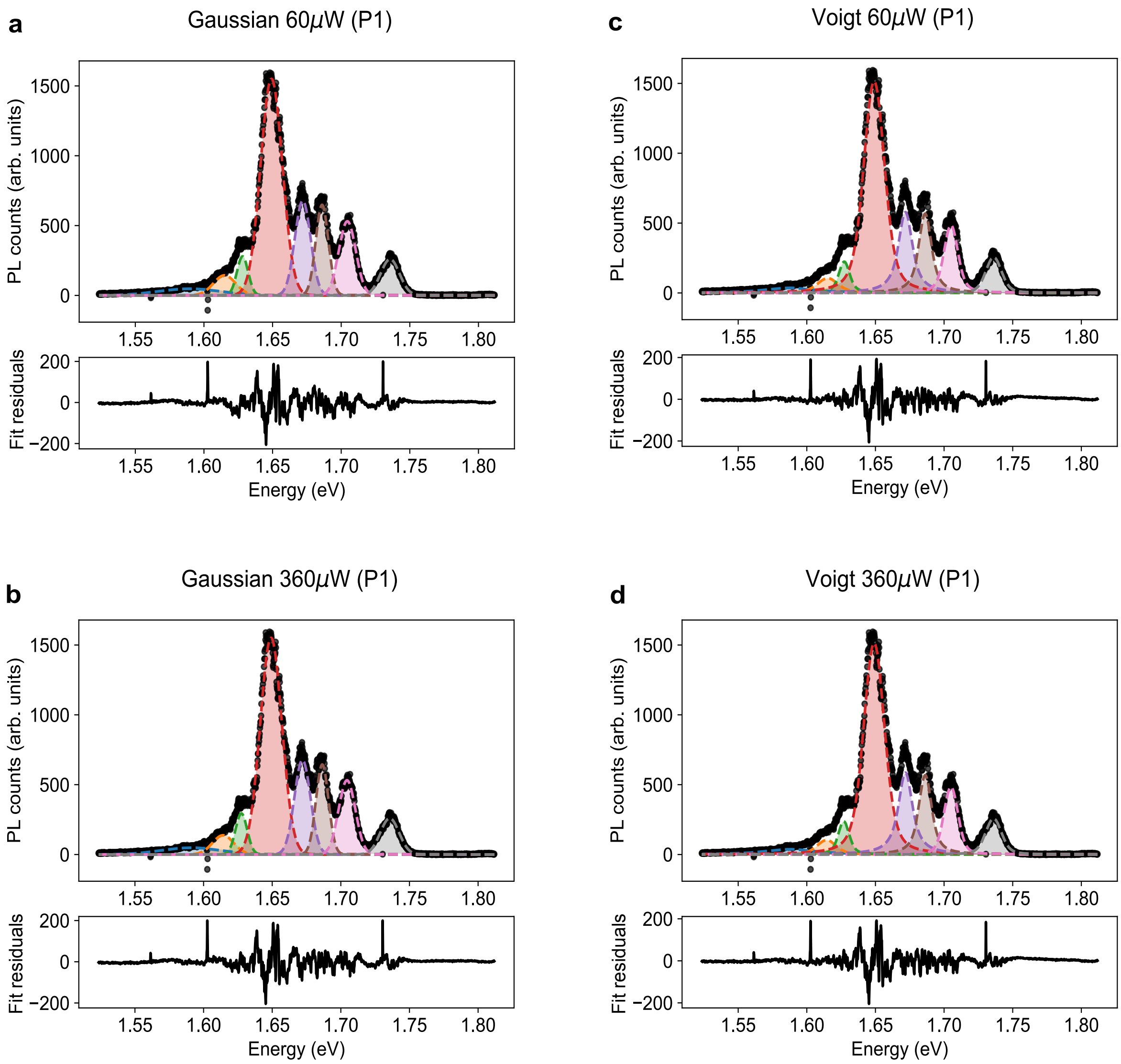

FIG. S8: **Comparison of Gaussian and Voigt peak fitting models for power-dependent photoluminescence spectra.** The emission spectrum of monolayer WSe_2 at 5 K is fit with a sum of eight peaks using either**a**,**b**, Gaussian or **c**,**d**, Voigt lineshape. The fits are shown for low (60 μW) and high (360 μW) excitation powers. For each panel, the raw data (black dots), the total fit (black line), and the individual fitted peaks (colored shaded areas) are shown. The lower panels display the fit residuals, calculated as the difference between the raw data and the total fit.

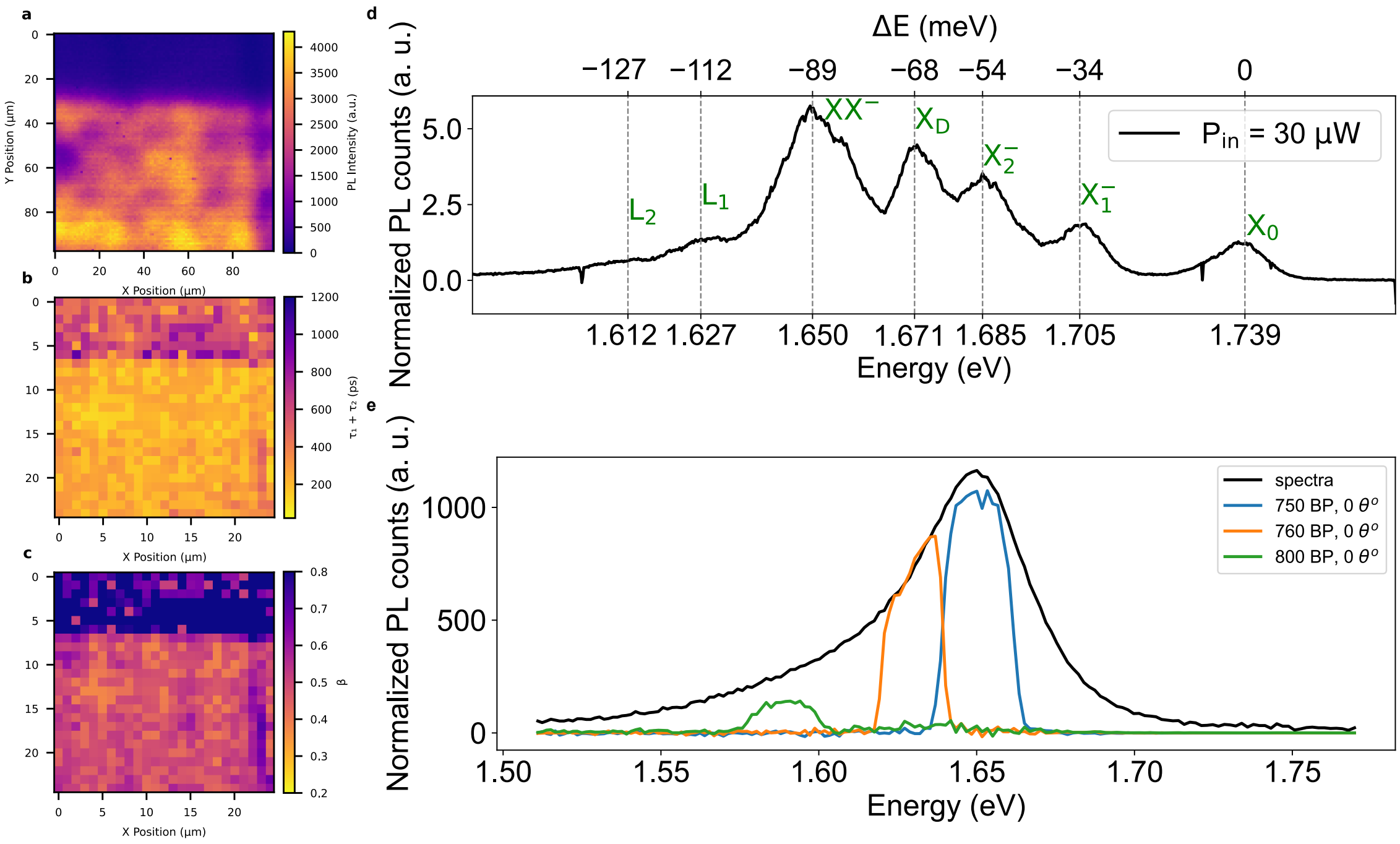

FIG. S9: **a**, Photoluminescence intensity scan. **b**, Map of the average lifetime ($\tau_{\rm avg}$). **c**, Map of the stretching exponent (β).**d**, PL spectrum with exciton peaks identified. **e**, PL spectrum (blue) of the sample (H33-S1) with transmission spectra of the bandpass filters used for energy-selective lifetime measurements: 750 nm (green, 0° tilt), 760 nm (orange, 0° tilt), and 800 nm (red) tilted by 25° to achieve a center wavelength of 780 nm.

S7. ROOM TEMPERATURE OPTICAL SETUP

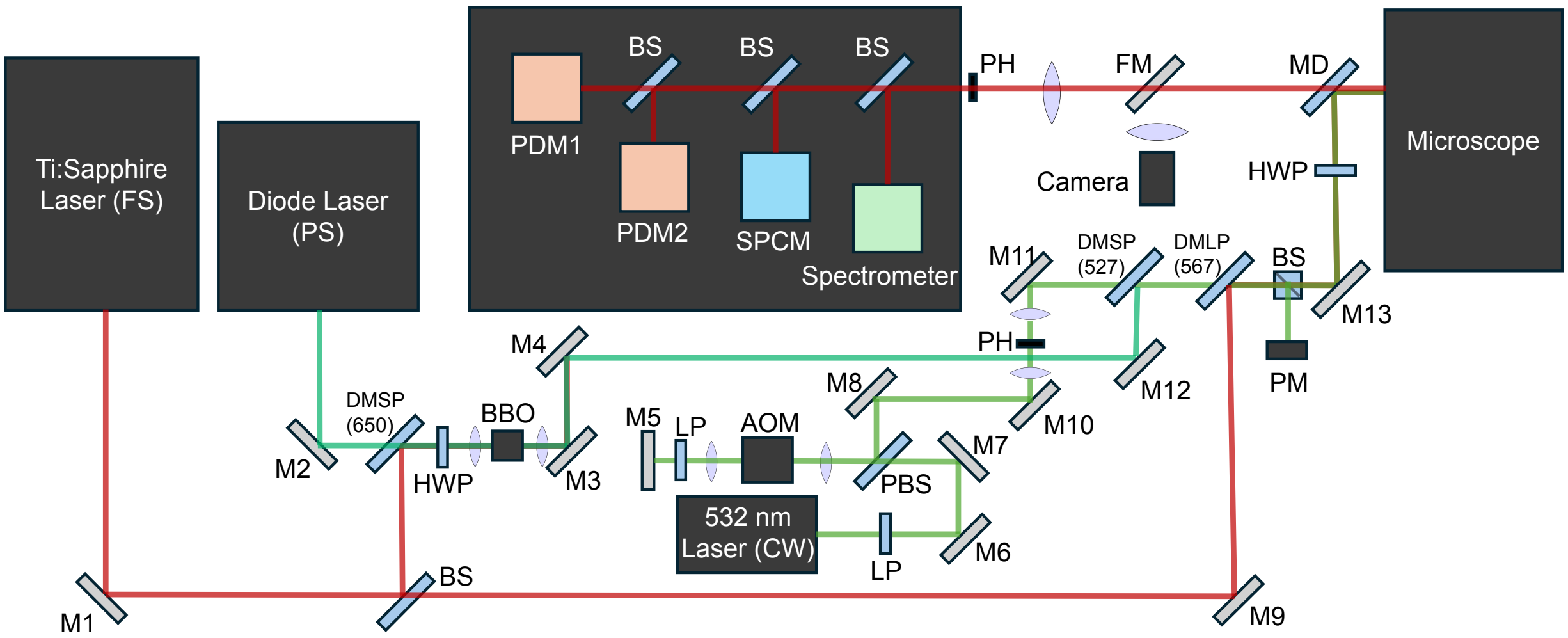

FIG. S10: **Schematic of the room-temperature optical setup.** The lasers include a Mai Tai HP DeepSee Ti:Sapphire laser (690–1040 nm, 150 fs pulse width, 80 MHz) and a continuous-wave 532 nm laser from RGB Photonics. A Becker & Hickl picosecond diode laser (515 nm, 40 ps, 80 MHz) is shown but was not used. A beta barium borate (BBO) crystal enables second harmonic generation (SHG) for tunable sources. Optical elements include silver mirrors (M1, M2, ...), acousto-optical modulator (AOM, Gooch & Housego 3350-199), linear polarizers (LP), half-wave plates (HWP), beam splitters (BS), polarizing beam splitters (PBS), a quarter-wave plate (QWP) and a 100 μm pinhole filter (PH). The QWP is mounted on a rotation stage (RS). Spectral filtering is done via dichroic mirrors: short-pass (DMSP), long-pass (DMLP), and main dichroic (MD). For resonant excitation at $\lambda = 750$ nm, the main dichroic was replaced with a broadband polarizing beam splitter. Photon counting was performed using SPADs (SPCM-AQRH, Excelitas; PDM series, Micro-Photon Devices). Spectra were collected using a QE65000 spectrometer (Ocean Insight).

Room-temperature measurements were conducted on a custom-built scanning confocal microscope, depicted in Fig. S10. The system is based on a Nikon Ti-U inverted microscope body. A Nikon LU Plan 100×/0.90 NA objective focuses the excitation laser and collects the resulting photoluminescence (PL). The sample is mounted on a three-axis piezo stage (Physik Instrumente, P-561 PIMars) controlled by an E-712 controller for precise positioning and raster scanning.

The optical path includes several key components. For continuous-wave (CW) excitation, a 532 nm laser (RGB Photonics) was used. For time-resolved measurements, a tunable Ti:Sapphire femtosecond laser (Spectra-Physics, Mai Tai HP; 690–1040 nm, 150 fs, 80 MHz) provided pulsed excitation, with a beta barium borate (BBO) crystal used for second harmonic generation when necessary.

The collected PL signal is directed through a main dichroic beamsplitter (Thorlabs, DMLP550L) and spectrally filtered by a 550 nm long-pass filter (Thorlabs, FEL0550) and a 750 ± 10 nm band-pass filter (Thorlabs, FBH750-10). In the case of spectrally filtered lifetime measurements (Main text, Fig. 4), the 750 BP was replaced with either the 550 LP, 760 BP, or 780 BP, as indicated. The light is then focused through a 100 µm pinhole for spatial filtering. For CW and scanning measurements, photons are detected with a single-photon avalanche diode (SPAD; Excelitas, SPCM-AQRH; 69% quantum efficiency at 650 nm). For lifetime measurements, a high-resolution SPAD (Micro-Photon Devices, PDM series; 30 ps resolution, 35% quantum efficiency at 650 nm) is used in conjunction with a Becker & Hickl SPC-150N time-correlated single-photon counting (TCSPC) module. Emission spectra were acquired using a QE65000 spectrometer (Ocean Insight). All hardware was integrated and controlled via a custom LabVIEW interface.

S8. EXTRACTION OF DOPING DENSITY

To determine the unintentional doping level in our monolayer WSe_2, we analyze the exciton and trion resonances observed in low-temperature photoluminescence (PL) spectra. The clear appearance of the negative trion (X^-) peak identifies the doping as n-type (electron doping). This is consistent with the fact that only electron doping stabilizes X^- states in WSe_2 [10].

From the PL spectra, we extract the exciton–trion energy splitting

$$\Delta E = E_{X^0} - E_{X^-} \approx 34 \text{ meV},$$

with $E_{X^0} = 1.739$ eV and $E_{X^-} = 1.705$ eV. In the high-density regime, where the electron Fermi energy exceeds the trion binding energy, the splitting is approximately given by [11]

$$\Delta E \approx E_{\mathrm{F}} + E_{\mathrm{bind}}^{X^-}.$$

Taking the trion dissociation energy as $E_{\mathrm{bind}}^{X^-} \approx$ 5-6 meV ($\sim$ 60-70 K), the measured splitting corresponds to a Fermi energy on the order of tens of meV. Using the 2D relation

$$E_{\mathrm{F}} = \frac{\pi \hbar^2 n}{m_e^*},$$

with the conduction-band effective mass $m_e^* \approx 0.3\, m_0$, this yields an electron density in the range

$$n_e \sim 10^{11}\text{–}10^{12} \text{ cm}^{-2}.$$

S9. NORMALIZED ENHANCEMENT CALCULATION

As mentioned in the main text, reported enhancement values in the literature span several orders of magnitude. Much of this variation arises from the specific normalization conventions employed in different experiments. For example, in plasmonic systems, the enhancement is often defined relative to the ratio between the excitation beam area and the nanoscale hotspot where the optical field is primarily localized. We refer to this normalization scheme as the "normalized" enhancement, enabling direct comparison with prior work. The normalized enhancement is defined as

$$\text{Normalized enhancement} = \text{Raw enhancement} \times \frac{A_{\mathrm{laser}}}{A_{\mathrm{mode}}}.$$

Here, A_{mode} denotes the effective mode area of the cavity, which is estimated to be $A_{\mathrm{mode}} \approx 30 \times 30 \text{ nm}^2$ based on geometrical estimates of the simulated near-field distributions. The laser spot area, A_{laser}, is estimated from the diffraction-limited beam waist,

$$w_0 \approx \frac{\lambda}{\pi \, \mathrm{NA}},$$

where $\lambda = 532$ nm and NA $= 0.9$, and is converted to an approximate laser spot area using $A_{\mathrm{laser}} \approx \pi w_0^2$. With this normalization, the raw enhancement factors from 10× to 14×, which we directly measured, yield mode-area-normalized enhancement factors ranging from 1200 to 1700.

[1] M. Newville, R. Otten, A. Nelson, T. Stensitzki, A. Ingargiola, D. Allan, A. Fox, F. Carter, and M. Rawlik, *Lmfit: Non-linear least-squares minimization and curve-fitting for python* (2025), URL https://doi.org/10.5281/zenodo.16175987.
[2] S. M. Sze and K. K. Ng, *Physics of Semiconductor Devices* (John Wiley & Sons, Hoboken, New Jersey, 2007), 3rd ed.
[3] H. A. Lorentz, *The Theory of Electrons and Its Applications to the Phenomena of Light and Radiant Heat* (Teubner, Leipzig, Germany, 1909).
[4] L. J. Prokopeva and A. V. Kildishev, Nanophotonics (2025), URL https://doi.org/10.1515/nanoph-2025-0044.
[5] D. De Sousa Meneses, G. Gruener, M. Malki, and P. Echegut, J. Non-Cryst. Solids **351**, 124 (2005).
[6] L. J. Prokopeva, S. Peana, and A. V. Kildishev, Comput. Phys. Commun. **279**, 1 (2022), URL https://www.sciencedirect.com/science/article/pii/S0010465522001321.
[7] D. Franta, J. Vohanka, and M. Cermak, *Universal dispersion model for characterization of thin films over wide spectral range* (Springer, Cham, Switzerland, 2018), pp. 31–82.
[8] V. N. Faddeyeva and N. M. Terentev, *Tables of Values of the Function* $w(z) = \mathrm{e}^{-z^2}\left(1 + \frac{2i}{\sqrt{\pi}}\int_0^z \mathrm{e}^{t^2}\,\mathrm{d}t\right)$ *for Complex Argument* (Pergamon Press, Oxford, UK, 1961).
[9] S. G. Johnson, *Faddeeva Package: A C++ library for the Faddeeva function*, http://ab-initio.mit.edu/faddeeva/ (2012), Last accessed on Sept. 6, 2025.
[10] Z. Li, T. Wang, Z. Lu, M. Khatoniar, Z. Lian, Y. Meng, M. Blei, T. Taniguchi, K. Watanabe, S. A. McGill, et al., Nano letters **19**, 6886 (2019).
[11] R. Perea-Causin, S. Brem, O. Schmidt, and E. Malic, Physical Review Letters **132**, 036903 (2024).
